# Advances in the Application of Sulfonated Poly(Ether Ether Ketone) (SPEEK) and Its Organic Composite Membranes for Proton Exchange Membrane Fuel Cells (PEMFCs)

**DOI:** 10.3390/polym16192840

**Published:** 2024-10-08

**Authors:** Xiang Li, Tengling Ye, Xuan Meng, Dongqing He, Lu Li, Kai Song, Jinhai Jiang, Chuanyu Sun

**Affiliations:** 1 Department of Applied Chemistry, School of Chemistry and Chemical Engineering, Harbin Institute of Technology, Harbin 150001, Chinaytl@hit.edu.cn (T.Y.); 2 School of Electrical Engineering and Automation, Harbin Institute of Technology, Harbin 150001, China; 23s106153@stu.hit.edu.cn (X.M.);; 3 Suzhou Research Institute, Harbin Institute of Technology, Suzhou 215104, China; 4 Institute of Advanced Technology, Heilongjiang Academy of Sciences, Harbin 150020, China; 5 Key Laboratory of Auxiliary Chemistry and Technology for Chemical Industry, Ministry of Education, Shaanxi University of Science and Technology, Xi’an 710021, China; 6 Shaanxi Collaborative Innovation Center of Industrial Auxiliary Chemistry and Technology, Shaanxi University of Science and Technology, Xi’an 710021, China

**Keywords:** SPEEK, PEMFCs, proton conductivity, fuel cell, composite membranes, ether ether ketone, Nafion, perfluorosulfonic acid membrane, polymer blending, inorganic-organic hybrid

## Abstract

This review discusses the progress of research on sulfonated poly(ether ether ketone) (SPEEK) and its composite membranes in proton exchange membrane fuel cells (PEMFCs). SPEEK is a promising material for replacing traditional perfluorosulfonic acid membranes due to its excellent thermal stability, mechanical property, and tunable proton conductivity. By adjusting the degree of sulfonation (DS) of SPEEK, the hydrophilicity and proton conductivity of the membrane can be controlled, while also balancing its mechanical, thermal, and chemical stability. Researchers have developed various composite membranes by combining SPEEK with a range of organic and inorganic materials, such as polybenzimidazole (PBI), fluoropolymers, and silica, to enhance the mechanical, chemical, and thermal stability of the membranes, while reducing fuel permeability and improving the overall performance of the fuel cell. Despite the significant potential of SPEEK and its composite membranes in PEMFCs, there are still challenges and room for improvement, including proton conductivity, chemical stability, cost-effectiveness, and environmental impact assessments.

## 1. Introduction

As society has developed, the massive consumption of traditional fuels has led to a series of serious environmental issues. Against the background of the continuous use of fossil fuels and the increasing demand for energy across the world, coupled with the energy crisis and worsening environmental pollution, seeking new energy technologies that are reusable, environmentally friendly, and that have high energy conversion efficiency is becoming an urgent problem [1]. Renewable energy sources, such as wind, solar and wave power, as well as other natural resources, are increasingly favored by scientists and are widely recognized as effective alternatives to fossil fuels [2,3]. However, due to inherent limitations in the intermittent and fluctuating nature of natural resources, their stability in energy supply and large-scale promotion have been limited. To solve this problem, research on new energy industries and key technologies that relate to energy storage is gaining an increasing amount of attention [4]. Among them, hydrogen energy, after a long period of technological accumulation, has made significant progress and has achieved relatively advanced applications in certain fields. Hydrogen energy holds significant potential in clean energy transition and is an effective pathway for achieving large-scale deep decarbonization in areas such as transportation, industry, and construction. Electrolysis of water for hydrogen production is not only one of the ways to obtain hydrogen energy, but also contributes to addressing the intermittency and variability of renewable energy sources. It helps to utilize excess natural energy from solar and wind power by storing electrical energy in the form of chemical energy, thus enhancing the efficiency of energy utilization [5,6].

As an efficient energy conversion device, the fuel cell (FC) plays an important role in the practical application of hydrogen energy. It can be said that fuel cells are one of the key technologies for achieving the “hydrogen economy” of the future [7,8]. Hydrogen fuel cells, serving as backup power sources or frequency regulation resources, can help to maintain grid stability during periods of supply and demand imbalance, thereby reducing reliance on fossil fuel power generation units. When there is an excess of renewable energy production, such as from solar and wind power, hydrogen is produced through electrolysis using water electrolyzers, which store the energy in the form of chemical one. During times of insufficient production, the stored hydrogen energy is converted back into electrical energy by the hydrogen fuel cells to meet the power demands during peak hours, achieving a balanced supply of energy. The peak-shaving and valley-filling function of hydrogen fuel cells can drive the development of the hydrogen industry chain, including hydrogen production, storage, transportation, and application. This process not only optimizes energy use but also contributes to the advancement of the hydrogen economy [9,10,11].

**Figure 1:**
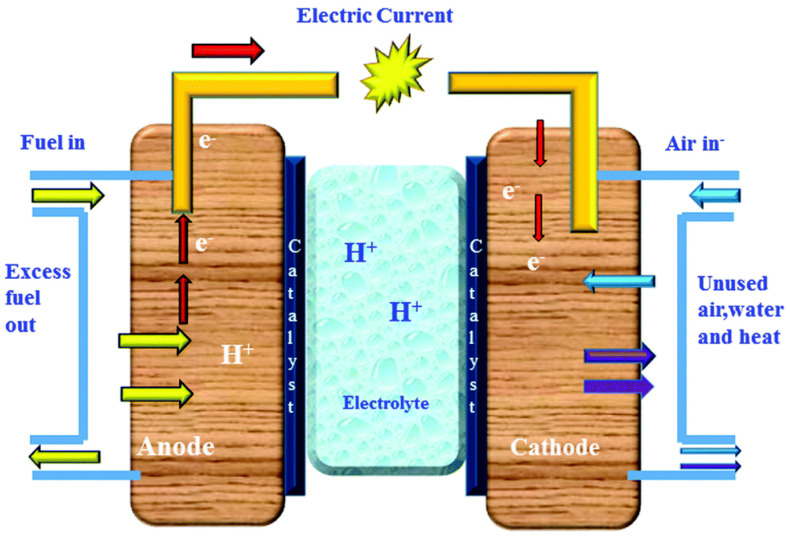
Fuel cell mechanism diagram [13]. Reproduced under terms of the CC−BY license [13]. Copyright 2022, *MDPI*.

Fuel cells are efficient and clean electrochemical power generation devices, which have been widely emphasized at home and abroad in recent years. Fuel cells are power generation devices that directly convert chemical energy in fuel into electric energy through electrochemical reactions. Classified by electrolyte, fuel cells generally include Proton Exchange Membrane Fuel Cells (PEMFCs), Phosphoric Acid Fuel Cells (PAFCs), Alkaline Fuel Cells (AFCs), Solid Oxide Fuel Cells (SOFCs), and Molten Carbonate Fuel Cells (MCFCs) [12]. A PEMFC consists of Bipolar Plates (BPs) with gas flow channels, Catalyst Layers (CLs) for catalyzing reactions, Gas Diffusion Layers (GDLs), and a Proton Exchange Membrane (PEM). Taking a hydrogen–oxygen fuel cell as an example, hydrogen is oxidized at the anode, and oxygen is reduced at the cathode. The mechanism of fuel cell is described in Figure 1. And the corresponding anodic and cathodic electrochemical reaction equations are as follows:

At the anode (oxidation reaction):

H_2_ → 2H^+^ + 2e^−^

At the cathode (reduction reaction):

2H^+^ + 1/2O_2_ + 2e^−^ → H_2_O

These reactions result in the production of water and the generation of electric current, making fuel cells a promising technology for clean and efficient energy production.

**Figure 2:**
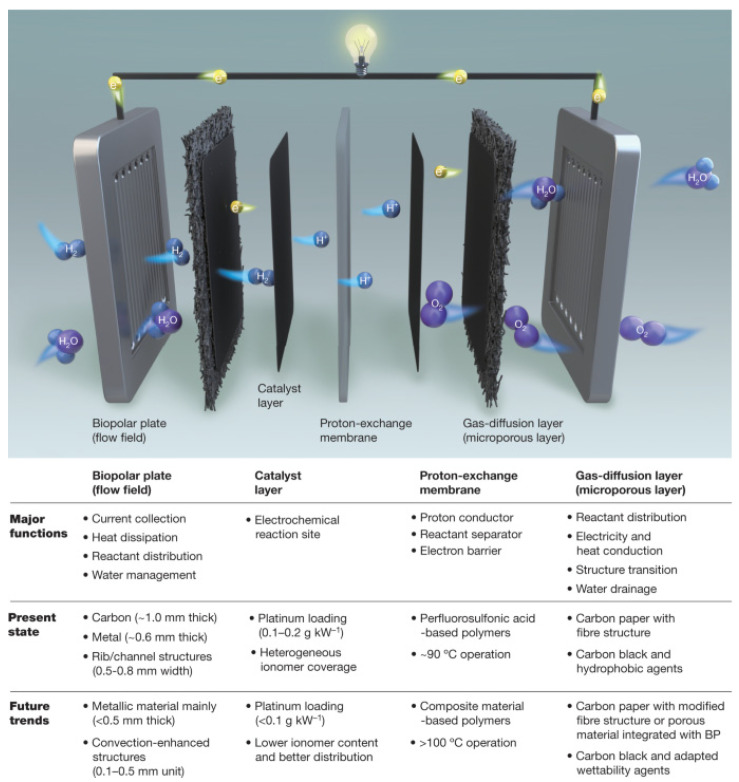
Overview of progressive improvements in PEMFCs to meet future high-power density requirements and a schematic explanation of the working principle [16]. Reproduced with permission from [16]. Copyright 2021, *Nature*.

As efficient energy conversion devices, PEMFCs show great potential in solving the unbalanced distribution of global green energy with their small size, energy-saving and environmental protection ability, short start-up time, high energy conversion efficiency, environmental friendliness, and simple hydrothermal management. PEMFCs achieve the goal of zero carbon emissions when using H_2_ as fuel, making them one of the most promising environmental protection power sources currently. Hydrogen fuel cells are typical proton exchange membrane fuel cells, which use hydrogen as the electrochemical reaction fuel, air as the oxidizing agent, and perfluorosulfonic acid proton exchange membrane as the electrolyte, and they generate electricity for the load through the electrochemical reaction [14]. Unlike other types of batteries, protons are transported through a proton exchange membrane, while electrons are transferred to the cathode via an external circuit. When hydrogen is used as fuel, an electrochemical reaction occurs at the anode, producing protons (H^+^) and electrons (e^−^). After oxygen is introduced, the protons, electrons, and oxygen react at the cathode to generate electrical energy. The water produced mainly exists in the form of liquid and is discharged from the cell along with the unreacted gas through the flow channels [12,15]. The overview of progressive improvements in PEMFCs and a schematic explanation of the working principle of PEMFCs is summarized in Figure 2.

PEM is one of the core components of PEMFCs, which has the functions of separating the cathode from the anode, providing a transport channel for H^+^, and acting as a barrier to gases. Therefore, the PEM must have sufficient stability and mechanical robustness to ensure that it will not be damaged by vibration and shock during operation. The development of PEMs with high proton conductivity, low fuel permeability, good chemical and thermal stability, strong mechanical properties, sufficient durability, and low costs is of major importance for the advancement of PEMFC technology.

**Figure 3:**
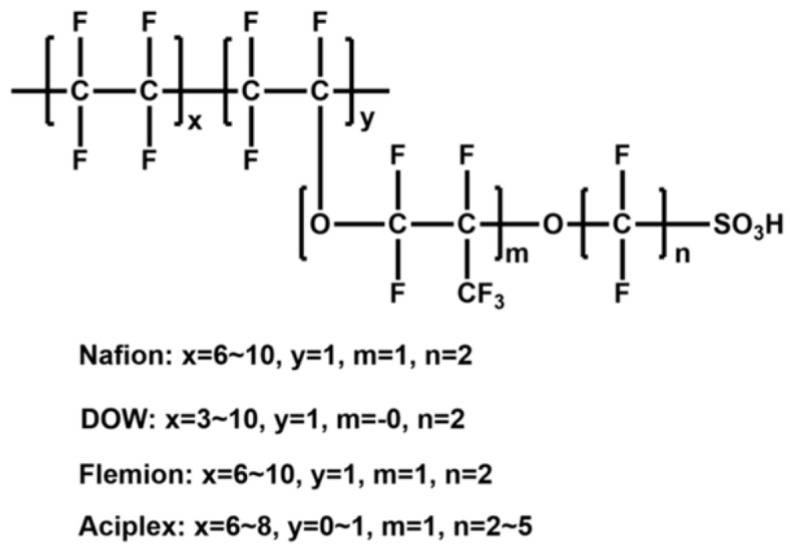
Perfluorinated PEM Structure [17]. Reproduced with permission from [17]. Copyright 2000, Elsevier Ltd.

At present, there are three main types of membrane materials on the market; namely, perfluorinated sulfonic acid (PFSA) membranes, partially fluorinated membranes, and non-fluorinated membranes. PFSA membranes have been developed as follows: (1) Nafion membranes from DuPont, including Nafion^®^ 115, Nafon^®^ 112, Nafion^®^ 117, Nafion^®^ 1035, etc.; (2) Aciplex^®^ membranes from the Asahi Chemical Company in Japan; (3) Flemion^®^ membranes from the Asahi Glass Company; (4) The Dow^®^ membranes from the Dow Chemical Company in the United States. (1) to (3) are long-branched PFSA PEMs, (4) being short-side-chain ones [17]. The structure of perfluorinated PEM is illustrated in Figure 3.

**Figure 4:**
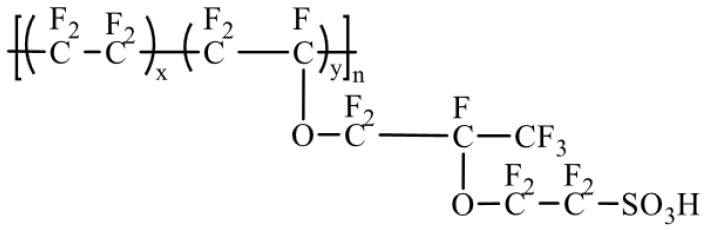
Chemical structure of Nafion.

At present, Nafion, a perfluorinated membrane produced by Chemours (USA), dominates the market, with the structure shown in Figure 4. The main chain of Nafion consists of carbon and fluorine atoms forming a polytetrafluoroethylene (PTFE) structure, while the side chain consists of ether-branched structures containing sulfonic acid groups(-SO_3_H). The main chain is hydrophobic, while the side groups contain hydrophilic -SO_3_H. This unique structure allows Nafion membranes to exhibit hydrophilic and hydrophobic phase-separated structures, resulting in membranes with high proton conductivity and good chemical stability [18,19].

However, Nafion membranes have some significant drawbacks in the application of PEMFCs, such as reduced proton conductivity at high temperatures due to water loss from the membrane material, higher fuel permeability, and high costs that limit their large-scale application in the market [20,21,22]. To reduce production costs and make up for the aforementioned shortcomings of Nafion membranes, more and more researchers are committed to developing enhanced PFSA membranes. For example, the Gore company prepared enhanced Gore-Select PFSA composite membrane by an impregnation–drying process of expanded PTFE and Nafion membrane, which greatly reduced the amount of PFSA resin used, resulting in a PEM with a performance that was superior to Nafion at a lower cost. Furthermore, researchers are committed to developing many non-fluorinated membranes as alternatives to Nafion [23], such as sulfonated poly(ether ether ketone) (SPEEK) [24,25,26,27], sulfonated polysulfone (SPSf) [28,29], polybenzimidazole (PBI) [30,31,32], sulfonated polyimide (SPI) [33,34,35], and poly (vinyl alcohol) (PVA) [36,37,38,39], etc.

Moreover, compared to Nafion membranes, non-fluorinated membranes offer several environmental advantages, which means they do not contain perfluorinated compounds such as per- and polyfluoroalkyl substances (PFAS) that have been associated with environmental and health concerns. In terms of environmental impact, non-fluorinated membranes have a lower global warming potential due to their lower carbon footprint during production. They are also more compatible with recycling processes, which can contribute to a circular economy. The advantages of non-fluorinated membranes in terms of environmental impact are also an important reason that attracts efforts directed at Nafion membranes replacement.

The durability of fuel cells is also closely related to the chemical structure of the membrane. During the long-term operation of fuel cells, due to the effects of start–stop cycles, wet–dry cycles, stress concentration, and alternating stress, PEMs are prone to undergo ‘swelling–shrinking’ cycles, leading to physical defects such as creep, cracking, and the formation of pinholes. These physical defects can exacerbate fuel permeation and reduce proton selectivity, ultimately leading to irreversible degradation and failure of the membrane. Therefore, the development of PEMs with good mechanical properties is crucial for both the performance and lifespan of fuel cell systems [40].

During the operation of fuel cells, oxygen that enters the three-phase interface where the PEM, catalyst, and GDL meet, and can react with protons that have been conducted to the cathode through the PEM from the anode to produce hydrogen peroxide (H_2_O_2_) as a side reaction. H_2_O_2_, under the catalytic action of certain metal ions, generates ·HO· and HOO· radicals, which will selectively attack the weak parts of the membrane, such as C-O-C and the H-containing terminal end groups (-CF_2_COOH), causing its irreversible chemical degradation, thereby reducing its proton conductivity and the service life of the fuel cell [41,42]. When radicals attack the C-F bonds in the side chains, causing them to break, H and F can also produce HF, a strong acid, which accelerates the degradation of the catalyst, reducing its loading and activity, and membrane, ultimately affecting its performance through reduction of ion exchange capacity (IEC), loss of proton conductivity, and thinning [43,44]. This has made the development of a fluorine-free PEM with excellent mechanical, chemical, and thermal stability a popular research direction.

Perfluorinated PEMs have several drawbacks, such as complex preparation processes, high cost, and poor fuel permeation resistance, which limits their further application [45]. In contrast, non-fluorinated PEMs, which are considered the most promising alternatives to Nafion^®^ membrane products, offer advantages such as a high cost-performance ratio and a more stable proton transport ability at high temperatures. Therefore, these membranes can maintain their water retention and mechanical properties over a wider temperature range [17,46]. The bonds of these proton exchange membranes are dominated by C-H ones, and to achieve strong stability, they are usually prepared by the sulfonation of aromatic polymers containing benzene rings in the main chain.

Polyether ether ketone (PEEK), as an aryl ether polymer, is an engineering plastic with excellent comprehensive properties. Due to its good thermal and chemical stability, as well its superior mechanical properties, PEEK is widely used in fields such as high-end machinery, nuclear engineering, aerospace, rail transit, military, and the petrochemical industry. PEEK itself is not electrically conductive; it only becomes conductive after the sulfonation process to form SPEEK, and the proton conductivity increases with the degree of sulfonation (DS) [47]. According to the literature, SPEEK with a sulfonation degree of 70% has a proton conductivity close to that of Nafion [48]. Therefore, SPEEK has shown great application potential in the field of fuel cells, especially in PEMFCs, and is considered one of the most likely candidates to replace Nafion as a fuel cell separator [49,50].

According to the targets set by the U.S. Department of Energy (DOE), the proton conductivity required for the practical application of PEMFCs should reach 100 mS cm^−1^ at 80 °C. It is well known that membranes with a high degree of sulfonation often have poor mechanical strength and severe swelling [51]. It has been reported that at a high DS (above 50%), SPEEK contains a larger mass fraction of sulfonic acid groups, and the prepared PEM can absorb more water, thus having higher proton transport performance. However, excessive water uptake will cause the chemical stability of the membrane to decrease rapidly. For instance, SPEEK with a DS of 87% can achieve a proton conductivity of 13.1 mS cm^−1^ at room temperature, but its tensile strength is only 24 MPa, while SPEEK with a DS of 67% has a proton conductivity of 7.5 mS cm^−1^ and a tensile strength of 44.7 MPa [52]. In addition, at a low DS (below 40%), the prepared PEM has better mechanical properties and thermal stability, but its proton conductivity will also be greatly reduced, falling below that of Nafion. Therefore, when the DS of SPEEK is within the range of 50–80%, the prepared PEM not only has a sufficient proton transport performance but also has good stability, exhibiting a better comprehensive performance [53,54,55].

In recent years, to achieve the optimal balance between proton conductivity and mechanical, chemical, and thermal stability for SPEEK membranes, many scientists have been committed to constructing a composite SPEEK membrane structure. The target is to improve the comprehensive stability of the membrane and the overall performance of the PEMFCs through simple doping or blending methods to prepare a SPEEK composite. A common modification approach is to use SPEEK as the base membrane and composite it with various inorganic particles or organic materials with excellent comprehensive performance to enhance the overall performance of the membrane material.

In recent years, SPEEK inorganic–organic hybrid membranes prepared from inorganic nanomaterials, such as zeolites, TiO_2_ [56], SiO_2_ [57], Fe_3_O_4_ [58], and others, have become increasingly popular. Meanwhile, the modification of PEMs for PEMFCs using carbon nanotubes (CNT) and metal–organic frameworks (MOF) has also become a hot topic of research in recent years [59,60].

Mawlood Maajal Ali’s team [61] prepared SPEEK with a sulfonation degree of 49.5%, and used carbon nanotubes after acidification with sulfuric and nitric acid to complex with it to obtain sPEEK/c-CNT composite membranes. At room temperature, the proton conductivity of the pristine SPEEK membrane was 23.9 mS cm^−1^, which increased with the addition of CNT content, reaching a maximum of 57 mS cm^−1^ at a CNT content of 0.12%.

Sivasubrmanian [62] prepared SP-CNT-FA composite membranes by introducing single-walled carbon nanotubes (SWCNTs) and fly ash (FA) into SPEEK (DS = 64%), which restricted the swelling behavior of the membrane and improved its mechanical and physical properties. The proton conductivity of the composite membrane with CNT and FA (0.034 S cm^−1^) was slightly higher than that of the SPEEK membrane (0.031 S cm^−1^). The tensile strength of SP-CNT-FA-8 reached 74.4 MPa, while the tensile strength of pristine SPEEK was only 54.6 MPa. During the single-cell test, the peak power density of the SP-CNT-FA-6 membrane at a load current density of 1625 mA cm^−2^ was 672 mW cm^−2^, while the peak power density of pristine SPEEK at 1500 mA cm^−2^ was 510 mW cm^−2^.

Zhiyu Dou and colleagues [63] incorporated nanoscale TiO_2_ particles into SPEEK (DS = 100%) membranes to fabricate composite membranes. The study found that the addition of TiO_2_ nano-inorganic particles increased the proton conductivity of the membranes. Specifically, the proton conductivity of SPEEK membranes containing TiO_2_ at 100 °C can reach 0.107 S cm^−1^, which is close to that of Nafion. Moreover, the incorporation of TiO_2_ particles enhanced the water uptake and retention rates of the composite membrane. Parisa Salarizadeh’s team [64] prepared sulfonated TiO_2_ nanoparticles and then grafted them with two monomers: sodium styrene sulfonate (SSA) and 2-acrylamido-2-methyl-1-propanesulfonic acid (AMPS), yielding sulfonated hybrid nanoparticles: PAMPS-g-TiO_2_ and PSSA-g-TiO_2_. These nanoparticles were introduced into SPEEK (DS = 68%) to fabricate nano–hybrid composite membranes. The grafted nanoparticles provided additional proton conduction sites, and compared to the SPEEK membrane, the proton conductivity of the nanocomposite membrane was enhanced further. Specifically, the composite membrane with nanoparticles had a proton conductivity 0.102 S cm^−1^, which is higher than that of the SPEEK membrane. Moreover, the mechanical and dimensional stability were also improved. Single-cell test results indicated that the membranes containing 7.5 wt% PAMPS-g-TiO_2_ and PSSA-g-TiO_2_ nanoparticles achieved maximum power densities of 283 mW cm^−2^ and 245 mW cm^−2^ at 80 °C, respectively.

Letícia G. da Trindade [65] first synthesized three types of ionic liquids (ILs): 1-butyl-3-methylimidazolium hydrogen sulfate (BMI.HSO_4_), 1-butylimidazolium hydrogen sulfate (BImH.HSO_4_), and triethylammonium propanesulfonate hydrogen sulfate (TEA-PS.HSO_4_). These ILs were then encapsulated within the metal–organic framework UiO-66 (Zr-MOF), and subsequently, the MOF encapsulating the ILs was combined with SPEEK to prepare composite membranes. The investigations indicated that the SMOF/TEA 2.5 membrane encapsulates TEA-PS.HSO_4_ exhibited the highest proton conductivity, ranging between 92 to 140 mS cm^−1^, while the proton conductivity of pristine SPEEK was 77 mS cm^−1^. The combination of CNT and inorganic materials with SPEEK provides a reference for the modification of PEM to improve its mechanical properties while maintaining relatively good proton conductivity.

Introducing a second phase with excellent physicochemical properties or constructing a crosslinking system is the most effective and practical method to enhance the comprehensive performance of SPEEK membranes. In addition, organic materials such as polyvinyl alcohol (PVA), polysulfone (PSU) [66], and polyvinylidene difluoride (PVDF) are also used as optional macromolecule components in blending to improve the mechanical and chemical stability of PEMFCs.

**Figure 5:**
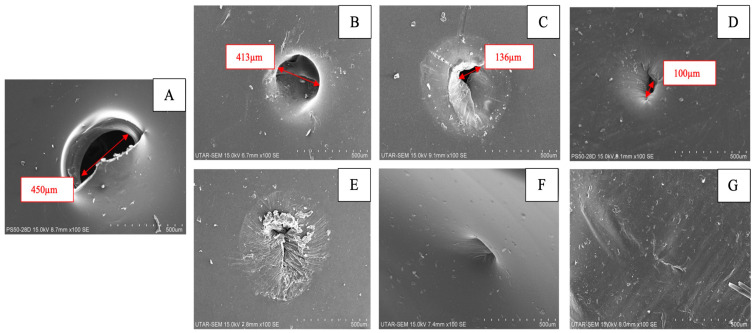
SEM image of (**A**) a damaged membrane, (**B**) SPEEK, (**C**) SP100, (**D**) SP30, (**E**) SP20, (**F**) SP10, and (**G**) SP0 after healing at 45 °C for 2 days [67]. Reproduced with permission from [67]. Copyright 2019, *Chemical Engineering Journal*.

Mae Hwa Tai and colleagues [67] blended SPEEK with PVA to prepare self-healing SPEEK/PVA composite membranes for direct methanol fuel cells (DMFCs). Due to the abundance of hydroxyl groups in PVA, reversible hydrogen bonds are formed with SPEEK. When the membrane experiences local defects due to stress during operation, the disrupted hydrogen bonds within the membrane will spontaneously revert to their original state. Additionally, the presence of water can cause PVA segments to move directionally to the defect area, where new hydrogen bonds are formed, restoring the continuity of the material [68]. The methanol permeability of the blended membrane is greatly reduced due to PVA’s low affinity for methanol and the interactions between SPEEK and PVA, which extends the lifespan of the DMFCs. However, since PVA is not conductive and lacks proton conduction sites, its addition reduces the proton conductivity of the composite membranes. The incorporation of PVA provides valuable experience and ideas for the research of self-healing and long-life PEMFCs, but it is still necessary to balance the relationship between the lifetime of the membrane and its comprehensive performance. Figure 5 depicts SEM images of the damaged and healed membranes.

Crosslinking is an effective method to restrict fuel diffusion and water uptake without sacrificing the good mechanical properties and integrity of the membrane. Dingbo Han et al. [69] prepared sulfonated SPEEK nanofiber felts with high DS, then the gaps were filled with acidic SPEEK. Finally, a heat treatment process was carried out to form crosslinks, while the SPEEK nanofiber felt did not. This preserved the sulfonic acid groups in the non-crosslinked areas, thus constructing long-range proton transport channels and improving the proton conductivity of the composite membrane. The composite membrane exhibited a high proton conductivity of up to 200 mS cm^−1^ and a peak power density of 485 mW cm^−2^. Xuehua Zhou and colleagues [70] modified SPEEK by incorporating PVDF grafted with polystyrene sulfonate (PSSA-g-PVDF) nanofibers. Experiments have demonstrated that the proton conductivity of the composite membrane was significantly enhanced after the nanofiber modification, reaching a maximum of 720.68 mS cm^−1^ and a power density of 470.52 mW cm^−2^, which is 5.5 times that of the original SPEEK.

The excessive addition of inorganic nanoparticles may increase the brittleness of the membrane. There is also a natural compatibility challenge between the organic and inorganic phases, which can lead to the cracking of the hybrid membrane due to phase separation. Ultimately, this can result in a decrease in the mechanical properties and chemical stability of the membrane, which is detrimental to the overall performance of the fuel cell system. Moreover, the manufacturing and processing of inorganic nanoparticle composite membranes can generate dust in the workshop, which is harmful to the health of the workers. Thermal cross-linking is an effective method to enhance fuel resistance and limit water uptake, offering valuable insights for the application of SPEEK and its modified composite membranes in PEMFCs. Polymers that are covalently crosslinked tend to become brittle in a dry state, which is a critical issue for fuel cell applications. The brittleness may be attributed to the rigidity of the covalent network [71,72]. Although the grafted polymer-modified composite membranes can substantially improve the proton conductivity of the membrane and the overall output performance of the cell, the high cost of this production method limits its large-scale commercialization. In contrast, organic co-blended membranes are prepared in a simple way, and the same solvent is selected to realize the simultaneous dissolution of SPEEK-based membranes and specific co-blended components. Moreover, the membrane fabrication process is simpler, avoiding the drawbacks of phase separation, so this paper pays more attention to the organic co-blended membranes of SPEEK.

This paper reviews the research progress of SPEEK and its organic composite blend membranes in fuel cells. By blending SPEEK material with organics that have excellent comprehensive properties, such as polysulfone polymers, fluoropolymers, and imidazole polymers, the advantages of SPEEK organic blend membranes compared to Nafion membranes are demonstrated. This aims to explore the commercial development of SPEEK as a substitute for Nafion and provides a summary and outlook for the application of SPEEK in PEMFCs.

## 2. Preparation of SPEEK Membranes

Polyether ether ketone (PEEK) is an engineering polymer material known for its excellent thermal stability, mechanical properties, and chemical resistance. However, its polymer chains are completely hydrophobic and do not possess proton conductivity, making it unsuitable for direct use in fuel cells. Therefore, sulfonation is commonly employed to introduce sulfonic acid groups into the side chains of PEEK, enhancing the polymer’s hydrophilicity and endowing it with proton conductivity [73]. SPEEK is typically prepared by sulfonating PEEK powder. The rigidity of the benzene ring structure results in weak electron adsorption capacity, which in turn leads to the acidity of the sulfonic acid groups in SPEEK being lower than that in Nafion, reducing the proton conductivity of the obtained materials [74]. To address this issue, the DS of SPEEK is often adjusted to ensure a high proton conductivity. However, as the DS increases, water uptake of SPEEK also enhances, making the material prone to uptake excessive water leading to an extremely high swelling ratio, which greatly reduces the mechanical properties and chemical stability of the membrane, thereby affecting the performance of the fuel cell. These drawbacks limit the commercial application of SPEEK membranes.

**Figure 6:**
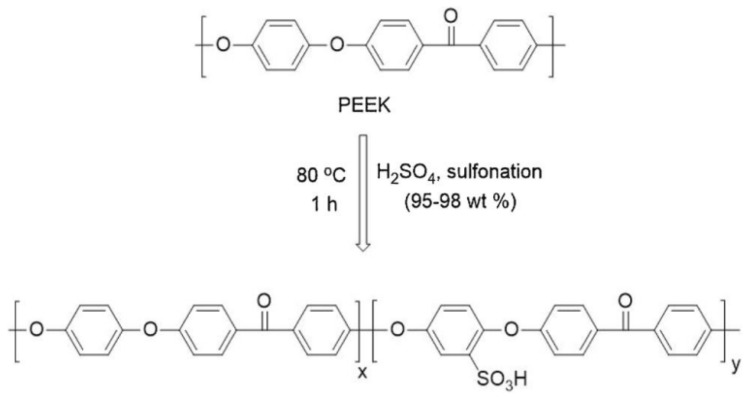
Sulfonation of PEEK [80]. Reproduced with permission from [70] Copyright 2021, *Physicochemical and Engineering Aspects*.

The main methods for sulfonation are direct polymerization and post-sulfonation. Direct polymerization refers to the method where small molecule sulfonated monomers are directly participating in the copolymerization process of the polymer. The DS is adjusted by controlling the amount of sulfonated monomer added. This approach can, to a certain extent, avoid side reactions such as polymer cross-linking and degradation that may be caused by sulfonation agents during the sulfonation reaction, while also offering a higher efficiency of sulfonation. Figure 6 shows the sulfonation of PEEK.

Prasad et al. [75] utilized two different sulfonated monomers: 1,3-propane sultone (0.5 mol) and 4,4′-difluorobenzophenone (0.5 mol), reacting them with bisphenol A (1.0 mol) to synthesize a sulfonated polyether ether ketone with sulfonic acid groups on the side chains. By adjusting the ratio of sulfonated and non-sulfonated monomers, the sulfonic groups could be quantitatively introduced into the polymer, allowing for control over the degree of sulfonation of SPEEK.

Huang et al. [76] synthesized SPEEK and prepared membranes using sulfonated 4,4′-difluorodiphenyl sulfone and 4,4′-difluorobenzophenone with bisphenol A as raw materials through direct polymerization. The ion exchange capacity (IEC) of the membranes ranged from 0.24 to 2.02 meq g^−1^, with water uptake rates between 2.26% and 26.45%. The swelling ratio of the membranes was between 1.71% and 15.28%. At room temperature, with a current density of 15 mA cm^−2^, the current efficiency reached was 58.31%.

The post-sulfonation method is a process of direct sulfonation of polymers using sulfonation reagents such as concentrated sulfuric acid [77] and chlorosulfonic acid [78], which directly introduce sulfonic acid groups (-SO_3_H) on the benzene ring through electrophilic reaction. The degree of sulfonation can be controlled by varying the reaction temperature and reaction time. This method offers a simple process for preparing SPEEK membranes. However, because the sulfonic acid groups introduced through post-sulfonation are located on the carbon atoms adjacent to the ether oxygen on the benzene rings, the electron-donating effect of the oxygen atoms can make the sulfonic acid groups more susceptible to hydrolysis and loss. Additionally, an excessively high degree of sulfonation can lead to the formation of by-products and the degradation of the polymer [79].

Lei Li et al. [81] used concentrated sulfuric acid to prepare SPEEK with different DSs through the post-sulfonation method. They studied the proton conductivity at various DSs, as well as the fuel permeability in methanol fuel cells. Their results showed that when the temperature was above 80 °C, the proton conductivity of membranes with a sulfonation degree of 39% and 47% both exceeded 10^−2^ S cm^−1^, approaching that of Nafion 115. Their methanol permeability was about an order of magnitude lower than that of Nafion 115. This also resulted in a higher open circuit voltage for the SPEEK membrane in single-cell tests (0.632–0.645 V) compared to that of the Nafion 115 membrane (0.595 V), indicating that it had a superior performance to Nafion 115.

Iulianelli et al. [82] carried out the sulfonation of PEEK using chlorosulfonic acid, resulting in SPEEK with a sulfonation degree ranging from 67% to 99%. Iulianelli posited that the proton transport and electrochemical performance of the membranes are influenced by the degree of sulfonation, uniformity, and membrane thickness. Poppe [83] also used chlorosulfonic acid as a sulfonating agent to prepare sulfonated polysulfone membranes with a DS between 53% and 100%. The thickness of these membranes was one-fourth that of Nafion 117. At 80 °C, the membranes exhibited a proton conductivity ranging from 0.11 to 0.23 S·cm^–1^, which was higher than that of Nafion.

## 3. SPEEK/Organic Composite Membrane

Sulfonated poly(ether ether ketone) is recognized as one of the most promising alternative materials for PEMs in fuel cell applications due to its good thermal stability, proton conductivity, mechanical strength, and low fuel permeability. The DS of SPEEK significantly affects its proton conductivity. By controlling the DS, the hydrophilicity and proton conductance of the membrane can be adjusted. However, an excessively high DS, while effectively enhancing the proton conductivity, may also lead to swelling, which in turn reduces the dimensional, chemical, and mechanical stability of the membrane. Meanwhile, when the temperature is too high (above 200 °C), the proton conductivity of the SPEEK membrane is significantly reduced due to the degradation of the sulfonic acid groups [84], which limits its widespread application in high-temperature fuel cells. To broaden the application of SPEEK membranes in fuel cell, SPEEK/organic composite membranes have been designed.

### 3.1. SPEEK/Polysulfone (PSF) Composite Membrane

Studies have found that blending sulfone polymers with SPEEK can significantly reduce the swelling ratio of the membrane and enhance its mechanical properties, as well thermal, dimensional, and chemical stability [85,86]. Due to the hydrophobic nature of sulfone groups and the acid–base interactions that can occur between sulfone substances and SPEEK, the water channels in the hydrophilic regions are restricted, thereby reducing the dissociation of the -SO_3_H groups. This phenomenon may generally lead to a reduction in the IEC and proton conductivity of the composite membrane compared to the pristine SPEEK membrane. However, because of the reduction in hydrophilic regions, the water uptake of the composite membrane is decreased, which limits the swelling and improves the mechanical stability and mechanical properties of the membrane [87,88]. In the composite membrane with sulfone substances, finding a balance between mechanical properties and proton conductivity is particularly important. Crosslinking is an effective method to improve the structural stability of the membrane, ensuring dimensional stability while suppressing methanol permeation of the polymer membrane in DMFC without overly sacrificing proton conductivity.

Srinivasan Guhan [89] fabricated SPEEK/PSF blend membranes with varying PSF contents. Their investigation results indicated that as the PSF content increased, the ion exchange capacity and the proton conductivity of the blend membranes decreased. However, the methanol permeability of the blend membranes also decreased, and their mechanical properties were enhanced. In single-cell tests, the PEMFC achieved a maximum power density of 400 mW cm^−2^. In DMFC single-cell tests, a maximum power density of 50 mW cm^−2^ was reached. Given that SPEEK/PSF exhibits good mechanical properties, mechanical stability, and durability, it has become one of the alternatives to Nafion as PEMs.

**Figure 7:**
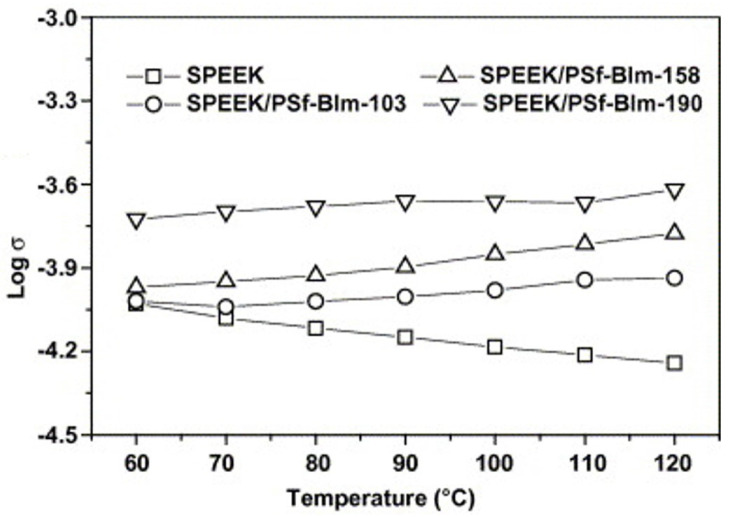
Variations of the proton conductivities of the SPEEK and SPEEK/PSf−BIm blend (3:1 weight ratio) membranes with temperature under anhydrous condition [90]. Reproduced with permission from [90]. Copyright 2006, *Electrochemistry Communications*.

Researchers have also investigated the blending of sulfone substances containing benzimidazole side groups with SPEEK. Similar to PBI, the N-sites in the imidazole side groups can transfer protons as proton conduction sites and are also suitable for high temperatures, enhancing the thermal stability of the membranes. Yongzhu Fu et al. [90] prepared PSf-BIm blend membranes by blending sulfone polymers containing benzimidazole side groups with SPEEK. The -SO_3_H in SPEEK can protonate the N in the imidazole moiety, allowing the composite membranes to conduct protons even at low humidity, with the proton conductivity increasing with temperature, which is described in Figure 7. In single-cell tests at 90 °C and 100 °C, the co-blended membranes showed better performance and lower polarization loss than pure Nafion and SPEEK membranes, and are also expected to be novel membranes capable of operating at high temperatures and low relative humidity.

W. Li [91] combined SPEEK with PSf featuring 4-nitrobenzotriazole (PSf-NBIm) side groups to fabricate SPEEK/PSf-NBIm composite membranes. At excessively high temperatures, as the temperature rises, the content of moisture decreases. Since water acts as a medium for proton conduction, this may lead to a gradual reduction in the proton conductivity of the pristine SPEEK membranes. However, since the nitrogen atoms on the benzimidazole ring can act as proton donors and acceptors, it can also enable the composite membranes to conduct protons, which led to an increase in the proton conductivity of the SPEEK/PSf-NBIm composite membranes. Concurrently, the insertion of imidazole side groups into the hydrophilic channel could also further inhibit the cross-permeation of methanol. In single-cell tests, under conditions of 80 °C and a 1 M methanol solution, the maximum power density of the single-cell with the SPEEK/PSf-NBIm composite membrane containing 2.5 wt% PSf-NBIm was 56 mW cm^−2^, which is 1.5 times that of the single-cell assembled with Nafion 115 (37 mW cm^−2^). In addition, it is noteworthy that the electrochemical stability test results showed that 4-nitrobenzimidazole showed a more friendly and mild nature with Pt catalysts and was less likely to poison the catalysts compared to imidazole under fuel cell operating conditions. This offers an advanced approach for extending the lifespan of catalysts, enhancing the proton conductivity, improving cell efficiency, and prolonging the durability of PEMFCs as well.

**Figure 8:**
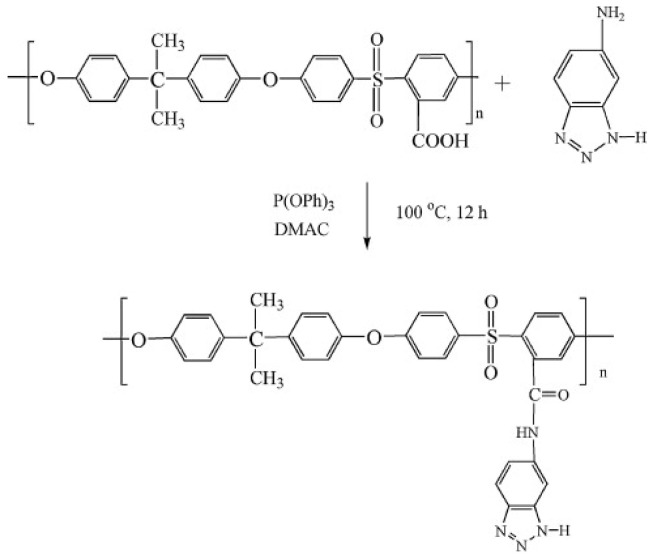
Synthesis scheme of polysulfone bearing 5-amino-benzotriazole side groups [92]. Reproduced with permission from [92]. Copyright 2010, *Journal of Membrane Science*.

Later, the team [92] prepared composite membranes of SPEEK with PSf bearing 5-aminobenzotriazole (PSf-Btraz) side groups. The synthesis scheme is illustrated in Figure 8. This membrane showed excellent performance in DMFC applications. The SPEEK/PSf-Btraz hybrid membrane containing 5 wt% PSf-Btraz exhibited higher proton conductivity and lower fuel permeability in DMFC compared to SPEEK and Nafion 115 ones. In cell testing, the SPEEK/PSf-BTraz blended membrane exhibited twice the maximum cell output power density of the Nafion 115 one at 80 °C and 1 M methanol feed.

**Figure 9:**
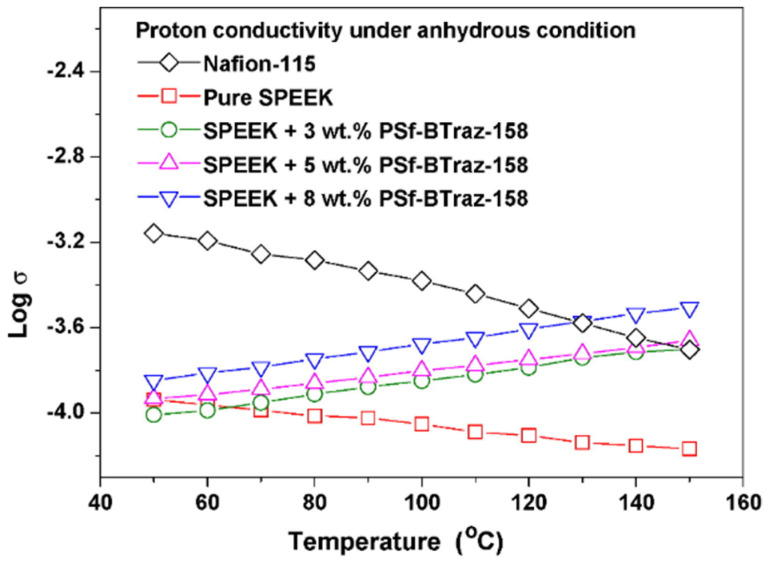
Comparison of the proton conductivities of the Nafion 115, pristine SPEEK, and SPEEK/PSf-BTraz (with various PSf−BTraz-158 contents) blend membranes under anhydrous conditions at various temperatures [92]. Reproduced with permission from [92]. Copyright 2010, Elsevier.

Because N on the side chain of BTraz can act as a proton donor and acceptor, it can also promote proton transfer under anhydrous conditions. The proton conductivity of Nafion 115 and SPEEK membranes decreased with the increasing temperature, but that of SPEEK/PSf-BTraz blended membranes enhanced with increasing temperature, which is shown in Figure 9. This helps to broaden the operating temperature range of PEMFC.

Han-Lang Wu [93] prepared a blended membrane of SPEEK (DS = 72%) and PES, and it was demonstrated that the proton conductivity of the SPEEK/PES blended membrane (0.034 S cm^−1^) was lower than that of Nafion 117 (0.08 S cm^−1^). But the methanol permeability of SPEEK/PES (14.2 × 10^−6^ cm^2^ s^−1^) was also much lower than that of Nafion 117 (3.2 × 10^−6^ cm^2^ s^−1^).

To improve the proton conductivity of SPEEK/PES blend membranes, researchers have used SPEEK with high DS compounded with PES to ensure their inherent proton conductivity. Daud [94] prepared blend membranes of SPEEK (DS = 80%) and PES. Their results showed that the incorporation of PES significantly enhanced the mechanical properties of the composite membranes with the addition of 5–20 wt% PES, increasing the tensile strength by about 11–51% and the elongation at break of 49–186% compared to the pristine SPEEK. The proton conductivity of the SPEEK/PES-5 composite membrane (7.181 mS cm^−1^) containing 5 wt% PES was close to that of the SPEEK one (8.14 mS cm^−1^). Meanwhile, the interaction between PES and SPEEK promoted the densification of the membranes. In the hydrogen–oxygen fuel cell test, the hydrogen permeability of the composite membranes was 0.655 × 10^−9^ cm^2^/scmHg, which was 63% lower than that of Nafion 117 and about 50–69% lower than that of SPEEK. During the single-cell test, the SPEEK/PES-5 membrane exhibited a performance comparable to the Nafion 117 one. At a temperature of 60 °C and a relative humidity (RH) of 100%, the power density and current density of the SPEEK/PES-5 were 99.29 mW cm^−2^ and 367.46 mA cm^−2^, respectively, while those of the Nafion 117 were 135.75 mW cm^−2^ and 421.08 mA cm^−2^. It is evident that the proton conductivity and cell performance of the SPEEK/PES-5 membrane are on par with the commercial Nafion 117, but with superior mechanical properties and a cost advantage. The novel combination of composition proposed by Daud provides a reference for the development of SPEEK membranes with good mechanical stability while ensuring adequate proton conductivity.

**Figure 10:**
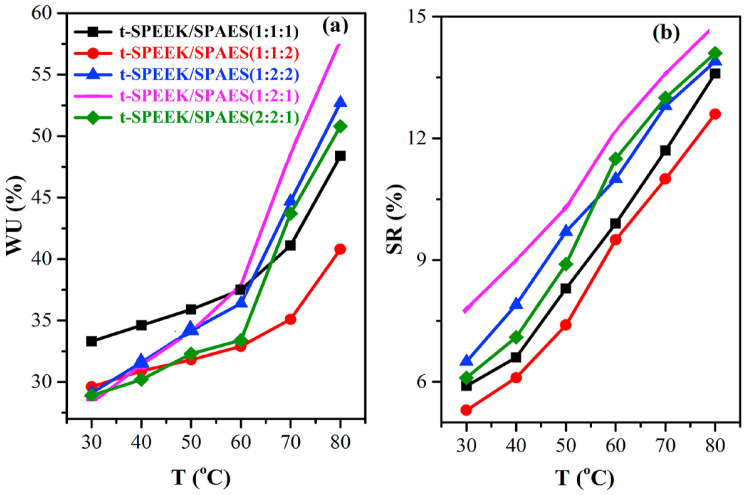
(**a**) Water uptake of the thermally-crosslinked SPEEK/SPAES membranes (**b**) swelling ratio of the thermally-crosslinked SPEEK/SPAES membranes [84]. Reproduced with permission from [84]. Copyright 2021, Elsevier.

**Figure 11:**
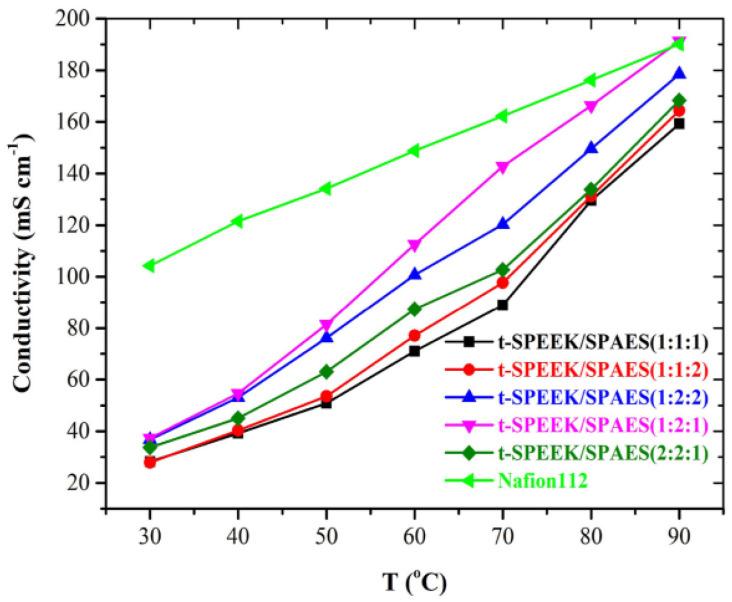
Proton conductivity of thermally cross−linked SPEEK/SPAES membranes and Nafion112 [84]. Reproduced with permission from [84]. Copyright 2021, Elsevier.

To further enhance proton conductivity, some researchers have sulfonated PES and then compounded it with SPEEK to prepare blending membranes. Alphonse Haragirimana et al. [84] used two types of SPEEK membranes with IEC values of 1.6 mmol g^−1^ and 2.0 mmol g^−1^, respectively, along with a sulfonated poly(aryl ether sulfone) (SPAES) with an IEC value of 2.0 mmol g^−1^, to fabricate blended membranes. Subsequently, thermal crosslinking was achieved through dehydrating reactions between the -SO_3_H groups and the active H atoms on the benzene rings. Thermal crosslinking significantly increased the dimensional stability of the membranes. Their results showed that the non-crosslinked membranes severely swelled at temperatures above 60 °C, while the crosslinked membranes exhibited a maximum swelling rate of only 15%, which is illustrated in Figure 10. This reduction in swelling was attributed to the consumption of -SO_3_H groups during the crosslinking process, which decreased the hydrophilic domains within the membrane structure. From Figure 11, the thermally crosslinked t-SPEEK/SPAES (1:2:1) membrane demonstrated the highest proton conductivity (191.3 mS cm^−1^), comparable to that of Nafion 112 (190.4 mS cm^−1^). In H_2_/O_2_ single fuel cell tests, a power density output of 665 mW cm^−2^ was achieved at 80 °C.

**Figure 12:**
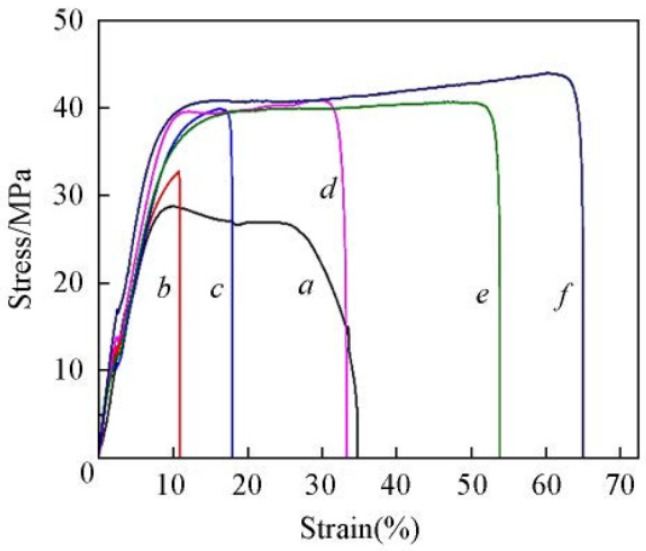
Mechanical strain–stress curves of SPEEK, SPFAES, and CMB membranes: a. SPEEK; b. SPFAES; c. CMB1; d. CMB2; e. CMB3; f. CMB4 [95]. Reproduced with permission from [95]. Copyright 2021, Higher Education Press.

Pu Yangyang et al. [95] prepared crosslinked SPEEK and partially fluorinated sulfonated poly(arylene ether sulfone) (SPEEK/SPFAES) cross-linked miscible blending (CMB) membranes by solution casting and simultaneous thermal crosslinking reactions. The IEC values of all membranes ranged from 1.21 to 1.51 mmol g^−1^, all of which were higher than that of Nafion 112. Studies have shown that these crosslinked blended membranes exhibit excellent performance in terms of mechanical strength, dimensional stability, chemical stability, and proton conductivity. Due to the introduction of crosslinking structures, the polymer underwent rearrangement of the main chains, and there was good compatibility between the two polymers, which enhanced the toughness of the CMB membrane and significantly improved its mechanical and antioxidant properties, effectively addressing the issue of membrane swelling. Mechanical strain–stress curves of these membranes is shown in Figure 12. The results showed that the tensile stress of the CMB membrane exceeded 40 MPa, with an elongation at break of up to 65%, which was significantly higher than that of pristine SPEEK and pristine SPFAES membranes.

**Figure 13:**
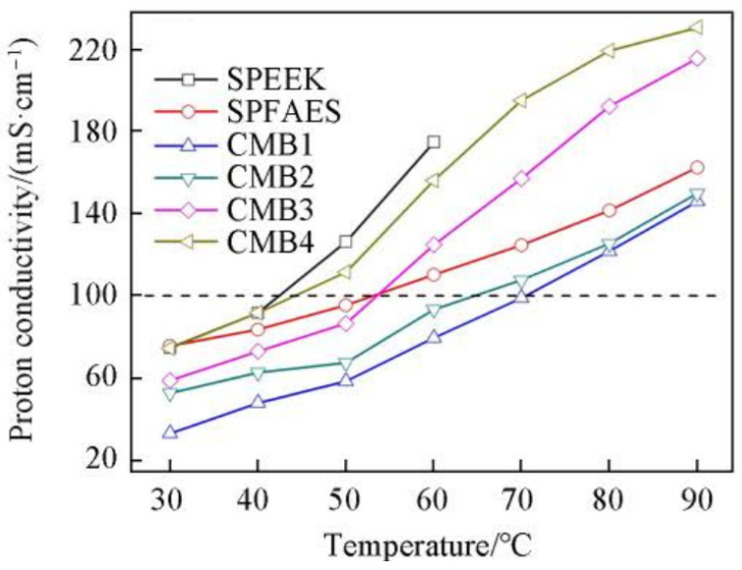
Proton conductivity of pristine SPEEK and CMB composite membranes at different temperatures [95]. Reproduced with permission from [95]. Copyright 2021, Higher Education Press.

**Figure 14:**
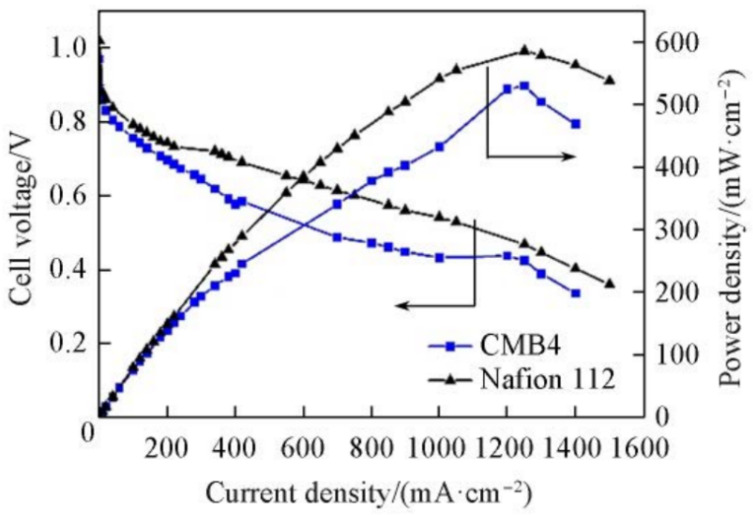
Polarization curves and power density profiles of single-cell test assembled with CMB4 and Nafion 112 membranes at 80 °C and 100% RH [95]. Reproduced with permission from [95]. Copyright 2021, *Chem. J. Chinese Universities*.

After the Fenton test, the composite membrane exhibited a mass loss of 0.8%, indicating a high level of antioxidant properties. Thanks to the good compatibility between SPEEK and SPFAES, and the crosslinking structure that facilitates the formation of hydrophilic proton channels, the CMB membrane can achieve a higher proton conductivity at a lower water uptake rate compared to the pristine SPEEK membrane. Test results showed that the proton conductivity of the CMB composite membrane could reach up to 190 mS cm^−1^ at 80 °C. The CMB4 composite membrane with a mass ratio of SPEEK to SPFAES of 4:1 could achieve a maximum proton conductivity of 219 mS cm^−1^ at 80 °C. A single-cell assembled with the CMB4 composite membrane demonstrated an open circuit voltage of 0.97 V during testing, which is comparable to the Nafion 112 membrane, indicating that the prepared CMB membrane has good fuel barrier capabilities. The maximum power density achieved for single-cell assembled with CMB membrane was 530.5 mW cm^−2^, which is slightly lower than that of Nafion 112. The proton conductivity of SPEEK, SPFAES, CMB membranes is illustrated in Figure 13 and polarization curves and power density profiles of single-cell test results are shown in Figure 14.

**Figure 15:**
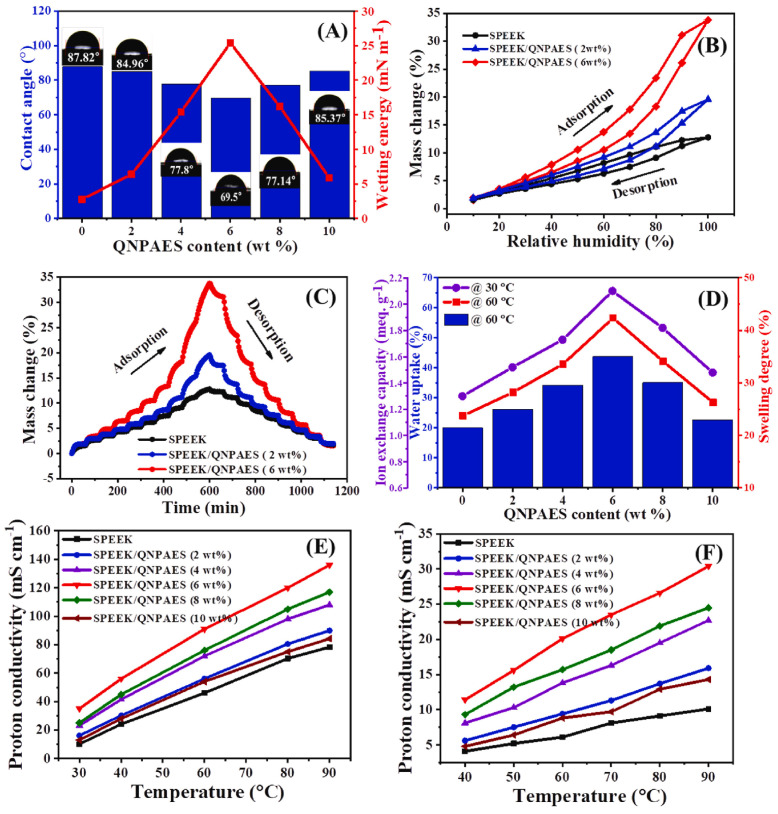
(**A**) Contact angle and wetting energy of pristine SPEEK and blending membranes at ambient temperature; (**B**) Water sorption/desorption properties with respect to humidity and (**C**) Water sorption/desorption properties with respect to time of pristine SPEEK and blending membranes at ambient temperature; (**D**) Water uptake and degree of swelling for pristine SPEEK and hybrids at 60 C and their IEC for pristine SPEEK and blending membranes at ambient temperature; (**E**) At different temperatures under 100% RH and (**F**) at different temperatures under 20% RH; proton conductivity plots of pristine SPEEK and blended membranes [96]. Reproduced with permission from [96]. Copyright 2023, Composites Part B: Engineering.

Ae Rhan Kim et al. [96] synthesized quaternized poly(aryl ether sulfone) (QNPAES), and then blended it with SPEEK to prepare SPEEK/QNPAES acid–base blend membranes via a solution casting method. Their results showed that these composite membranes exhibited excellent electrochemical performance and durability at a relatively low humidity (RH = 20%). Due to the ionic bonds formed between the -NR_3_(OH) ionic groups in the QNPAES polymer and the -SO_3_H groups in SPEEK that promoted proton migration, the proton conductivity of the composite membrane was significantly enhanced. At 90 °C and 100% RH, the proton conductivity of SPEEK/QANPAES (6 wt%) could reach up to 136 mS cm^−1^, which is about 1.7 times that of the original SPEEK membrane; at 20% RH, the proton conductivity could reach 30.4 mS cm^−1^, which is almost three times that of the original SPEEK membrane under the same humidity conditions. The ion exchange capacity, contact angle, moisture adsorption/desorption, wetting energy, and swelling degree of pristine SPEEK and blending membranes are shown in Figure 15.

The -NR_3_(OH) groups in QANPAES and the -SO_3_H groups in SPEEK form strong acid–base interactions, significantly enhancing the durability of the membrane material. At 60 °C and 100% RH, a H_2_/O_2_ PEMFC containing the SPEEK/QANPAES (6 wt%) composite membrane achieved a peak power output of 0.56 W cm^−2^ at a current density of 1.18 A cm^−2^, which is 2.07 times that of original SPEEK and 1.3 times that of Nafion 212. At 60 °C and 20% RH, the fuel cell assembled with the composite membrane reached a peak power density of 0.15 W cm^−2^ at the current density of 0.431 A cm^−2^, which is 3.19 times that of the original SPEEK and 2.24 times that of Nafion 212.

The properties of SPEEK/PSF composite membranes are summarized in Table 1.

### 3.2. SPEEK/Imidazole Composite Membrane

Polybenzimidazole (PBI) refers to a class of polymers that contain benzimidazole aromatic heterocycles in their main chain structure. They possess excellent thermomechanical properties, making them suitable for high-temperature operating applications, such as high-temperature proton exchange membrane fuel cells (HT-PEMFCs), high-temperature protective clothing materials, and heat-resistant coatings [97]. HT-PEMFCs have attracted much attention in recent years, operating at temperatures between 130 °C and 200 °C with fast molecular reaction kinetics, while the presence of water in the form of vapor simplifies water and thermal management. HT-PEMFCs require less catalyst activity than low-temperature PEMFCs, and demonstrate higher power densities compared with conventional fuel cells [98,99,100]. PBI doped with phosphoric acid (PA) and its composite membranes with SPEEK are widely used in HT-PEMFCs. They exhibit good proton conductivity without external humidification at high temperatures, excellent chemical resistance, higher glass transition temperatures, outstanding thermal stability, and superior mechanical properties [101,102,103].

Composite membranes of SPEEK and PBI are also gaining an increasing amount of attention. SPEEK and PBI exhibit good compatibility, and when the PBI content is below 15 wt%, there is no microphase separation in the blend membranes. PBI is distributed relatively uniformly within the SPEEK matrix, with almost no aggregation occurring. There is an acid–base interaction between the sulfonic acid groups in SPEEK and the imidazole groups in PBI, which can reduce the dependence of the PBI membrane material on the amount of phosphoric acid doping. Moreover, the formation of a hydrogen bonding network can suppress the movement of polymer chains, thereby enhancing the stability of SPEEK [104,105]. The integration of SPEEK with PBI to form hydrogen bonds and chemical bonds hinders the oxidation of free radicals, thereby enhancing the oxidative stability of the membrane [106,107]. It is well known that membranes with high water uptake rates tend to be brittle and soluble in water at high temperatures, exhibiting poor stability. SPEEK is hydrophilic, with a high water uptake rate, and can dissolve in hot water above 40 °C. The formation of ionic crosslinks between SPEEK and PBI significantly reduces the water uptake rate of SPEEK and allows it to remain stable in boiling water, greatly improving its stability. Meanwhile, the interaction between the sulfonic acid groups and amine groups also affects the connectivity of the hydrophilic regions, which in turn affects the transport properties of the membrane. The addition of PBI reduces the hydrophilic regions of the membrane, which also lowers its water uptake rate and proton conductivity. Generally speaking, PBI itself is not conductive, thus the proton conductivity of SPEEK/PBI composite membranes will be lower than that of SPEEK, which is also related to the decreased amount of free sulfonic acid groups in the composite membrane. The -SO_3_H in SPEEK transfers protons to the N atom of the imidazole group, reducing the number of free -SO_3_H groups and the number of proton conduction sites, and thus lowering the proton conductivity.

In the blending research with PBI, SPEEK with a higher DS and PBI form a composite membrane with excellent compatibility through acid–base interactions. The mechanical properties and chemical stability of this kind of composite membrane are significantly improved [108]. Therefore, finding a balanced state between proton conductivity and mechanical performance to ensure high overall membrane performance and comprehensive cell output performance is an important research objective [109].

Sivakumar Pasupathi et al. [110] mixed SPEEK solution with PBI solution uniformly and prepared SPEEK/PBI composite membranes via a solution casting method. The proton conductivity of the composite membrane was 0.0046 S cm^−1^, significantly lower than that of Nafion (0.016 S cm^−1^), but its thickness value (55 µm) was lower than that of Nafion 117 (170 µm). The power density of the fuel cell assembled with the SPEEK/PBI composite membrane was twice as high as that of Nafion 117 at 60 °C, which may be related to the weaker methanol-blocking ability of SPEEK, leading to a low permeability of methanol through the composite membrane. However, the paper did not test the mechanical properties and fuel crossover of the membrane.

To maintain a high proton conductivity value, Haiqiu Zhang [104] prepared highly sulfonated SPEEK (DS = 110%) and combined it with PBI to obtain SPEEK/PBI composite membranes. After the introduction of PBI, because of the acid–base interactions between the sulfonic acid groups and the amine groups, the water uptake and proton conductivity of the composite membranes were reduced compared to pure SPEEK. However, the proton conductivity of all membranes was above 100 mS cm^−1^, meeting the requirements for use in low-temperature PEMFCs. At 80 °C, the proton conductivity of the SPEEK/PBI-5 composite membrane (containing 5 wt% PBI) was 80 mS cm^−1^, slightly lower than that of the original SPEEK membrane at the same temperature (97 mS cm^−1^). But also due to the acid–base interactions within the membrane structure, the structure became denser, limiting the water uptake and swelling effect, as well as methanol permeation in DMFCs, which exhibits better dimensional stability and lower fuel permeability. The methanol permeability of SPEEK was 14.8 × 10^−7^ cm^2^/s, and when 20 wt% PBI was added to the composite membrane, the methanol permeability was 0.3 × 10^−7^ cm^2^/s, significantly lower than that of Nafion 117 (13 × 10^−7^ cm^2^/s) [111].

**Figure 16:**
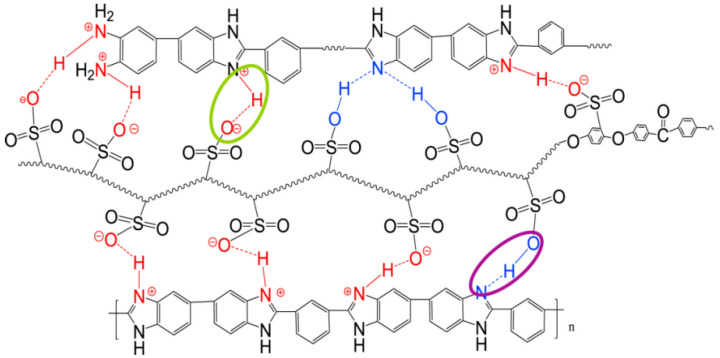
The structure of SPEEK/PBI composite membranes [112]. Reproduced with permission from [112]. Copyright 2016, Elsevier Ltd.

Song Mingfeng [112] and colleagues prepared highly sulfonated SPEEK and combined it with PBI to create ionically crosslinked SPEEK/PBI acid–base composite membranes with good compatibility. The structure of SPEEK/PBI composite membranes is shown in Figure 16. Due to the intense ionic−crosslinking between -SO_3_H and double bond of C and N atom, as is shown in the circle of Figure 16, these composite membranes exhibited better heat resistance and proton conductivity compared to original SPEEK membranes. The formation of ionic crosslinking and hydrogen bonding between SPEEK and PBI resulted in promoted heat resistance in the composite membranes with an increase in PBI content. The proton conductivities of the SPEEK/PBI (10 wt%), SPEEK/PBI (15 wt%), and SPEEK/PBI (20 wt%) composite membranes were 0.257 S cm^−1^, 0.292 S cm^−1^, and 0.1985 S cm^−1^, respectively, with corresponding heat resistance temperatures of 110 °C, 130 °C, and 170 °C.

Concurrently, the acid–base interactions also resulted in a dense membrane structure, which significantly improved the mechanical properties. With the increase of PBI content, the tensile strength of SPEEK was greatly enhanced. The strengths of the SPEEK/PBI (20 wt%) and SPEEK/PBI (30 wt%) composite membranes reached 45.06 MPa and 50 MPa, respectively. The SPEEK/PBI also exhibited good resistance to methanol, with a permeability of 1.21 × 10^−8^ cm^2^ s^−1^ for SPEEK/PBI (20 wt%) at 30 °C, significantly lower than that of pure SPEEK (5.94 × 10^−8^ cm^2^s^−1^). At a higher temperature (70 °C), the methanol permeability of SPEEK/PBI (20 wt%) was 3.96 × 10^−8^ cm^2^ s^−1^, lower than that of pure SPEEK (1.29 × 10^−8^ cm^2^ s^−1^) and two orders of magnitude lower than that of Nafion 117 (1.0 × 10^−6^ cm^2^ s^−1^), which provides significant advantages in DMFC applications. In a 24-h Fenton test, the weight loss of pure SPEEK was 18.68%. With the increase of PBI content, the hydrogen bonding effect was enhanced, and the antioxidant properties of the composite membranes were also gradually improved, with the weight loss in the Fenton test decreasing accordingly. The composite membrane containing 40 wt% PBI had a weight loss of only 8.21%. The incorporation of PBI provides an important solution for the preparation of PEMs with excellent comprehensive performance for applications in HT-PEMFCs.

To further enhance the comprehensive performance of SPEEK/PBI composite membranes, many researchers are also committed to finding other organic/inorganic fillers to modify the composite membranes, such as graphene, graphene oxide (GO) [113], and ionic liquids (IL) [114]. Various inorganic materials possess hygroscopicity and water retention, and membranes composited with inorganic materials help to bind water together, providing internal hydration for proton conduction. The surface of GO has carboxyl groups that can facilitate proton hopping, and the increased number of proton conduction sites can enhance the proton conductivity of membranes [115].

**Figure 17:**
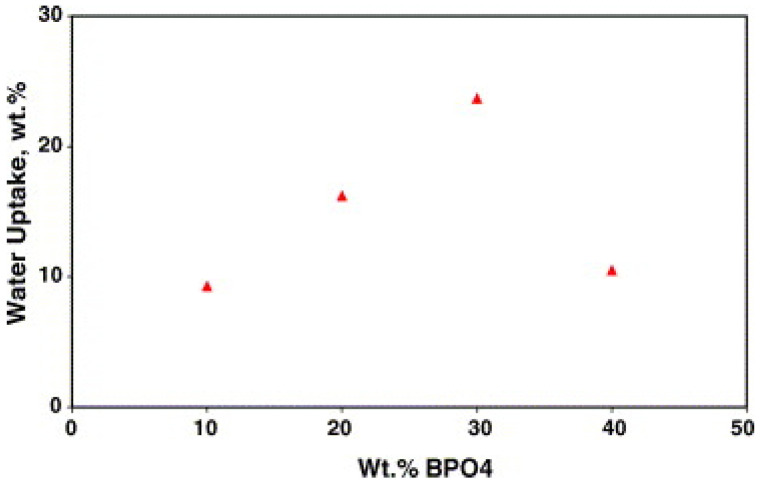
Water uptake of SPEEK/PBI composite membranes containing BPO_4_ [116]. Reproduced with permission from [116] Copyright 2005, Elsevier Ltd.

**Figure 18:**
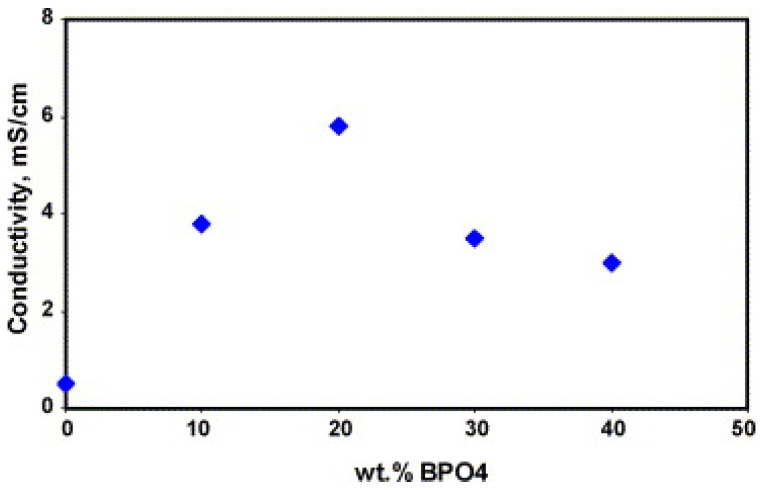
Proton conductivity of composite SPEEK/PBI blend membranes containing BPO_4_ [116]. Reproduced with permission from [117] Copyright 2005, Elsevier Ltd.

S. M. Javaid Zaidi [116] prepared SPEEK/PBI composite membranes by doping boron phosphate (BPO_4_) to improve proton conductivity. Infrared test results indicated that BPO_4_ exists in the composite membrane in solid form, and the BPO_4_ itself can conduct protons and retain water at high temperatures, with a proton conductivity of 6 × 10^−2^ S cm^−1^. The author prepared composite membranes of SPEEK/PBI (10 wt%) with different contents of BPO_4_ and found that, within a certain range, both proton conductivity and water uptake increase with the increase of BPO_4_ content. The proton conductivity of the composite membrane without BPO_4_ was 0.47 mS cm^−1^, while that of the composite membrane containing 20 wt% BPO_4_ was 5.9 mS cm^−1^. Interestingly, the addition of BPO_4_ reduced the water content of the composite membrane, as is shown in Figure 17. The water content of the SPEEK/PBI mixture was 30%, while the composite material containing 20 wt% BPO_4_ was only 16%, but it enhanced the proton conductivity of the composite membrane, as is shown in Figure 18. This may be due to BPO_4_ enhancing the acidity of the sulfonic groups in the polymer, leading to new interfacial polymer–particle properties that facilitate proton conduction. The proton conductivity of the SPEEK/PBI membrane with the addition of BPO_4_ is comparable to that of Nafion, approximately 100 mS cm^−1^, but it has better chemical stability than Nafion. It can be concluded that incorporating inorganic conductive materials into SPEEK/PBI membranes is one of the effective methods to promote proton conductivity.

**Figure 19:**
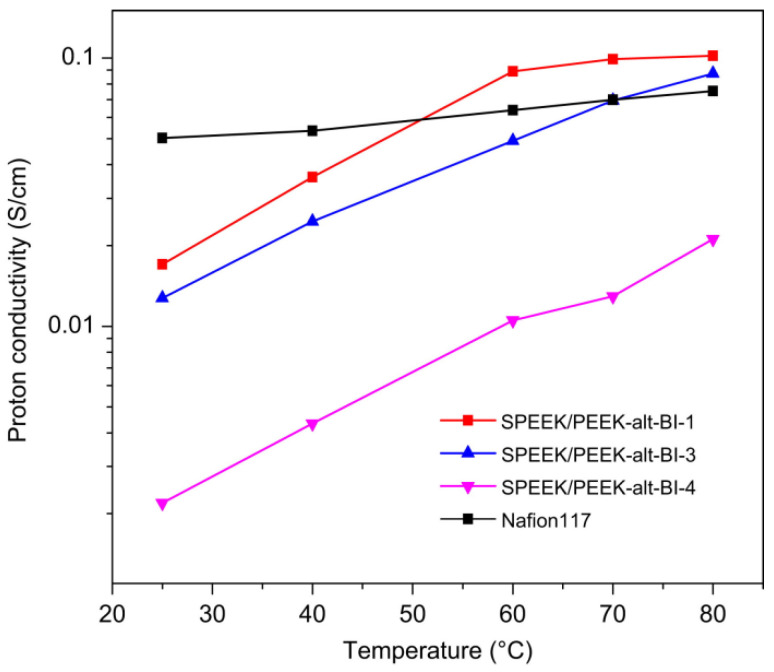
The proton conductivity of blended membranes and Nafion117 [117]. Reproduced with permission from [117] Copyright 2012, Elsevier.

Jianli Wang [117] introduced poly(ether ether ketone-alt-benzimidazole) (PEEK-alt-BI) into SPEEK with high DS (86.5%) to prepare an acid–base blended membrane with good compatibility. The acid–base interactions between the sulfonic acid groups and the benzimidazole groups enhanced chemical resistance to hydroxyl radicals and formed a polymer network complex, which endowed the membrane with good chemical stability and mechanical properties. The incorporation of PEEK-alt-BI reduced the size of the hydrophilic ionic clusters. However, when doped with the 15 wt% PEEK-alt-BI, the SPEEK/PEEK-alt-BI blended membrane exhibited a proton conductivity of 87 mS cm^−1^ at 80 °C, which was higher than that of Nafion 117 measured under the same conditions. The proton conductivity of blended membranes and Nafion117 was shown in Figure 19.

Simultaneously, mechanical property testing was conducted on the prepared membranes, and the results indicated that as the incorporation of PEEK-alt-BI (5–20 wt%) increased, and the degree of ionic cross-linking also increased, leading to a closer molecular arrangement. This resulted in an increase in both the tensile strength and tensile modulus of the blend membrane. The tensile modulus and tensile strength of the original SPEEK was 624 MPa and 21.5 MPa, respectively, while the corresponding values for the blend membrane were higher than those of SPEEK, reaching a maximum of 753.4 MPa and 34.6 MPa. This composite membrane exhibited a better overall advantage over the Nafion membrane at temperatures above 70 °C.

**Figure 20:**
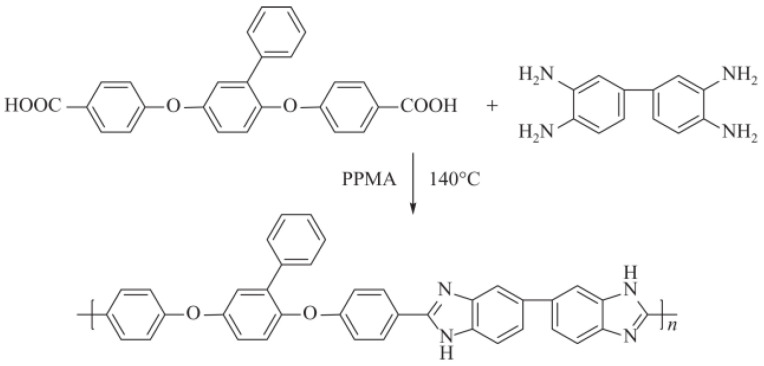
Synthesis of phenylated polybenzimidazole [118]. Reproduced with permission from [118] Copyright 2013, SAGE Publications Ltd.

**Figure 21:**
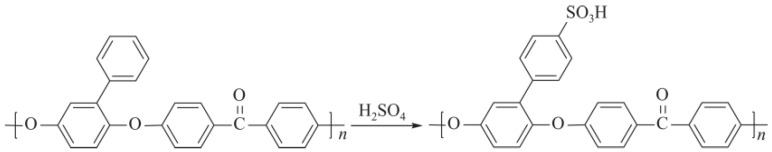
Synthesis of phenylated-sulfonated poly(ether ether ketone) [118]. Reproduced with permission from [118] Copyright 2013, SAGE Publications Ltd.

**Figure 22:**
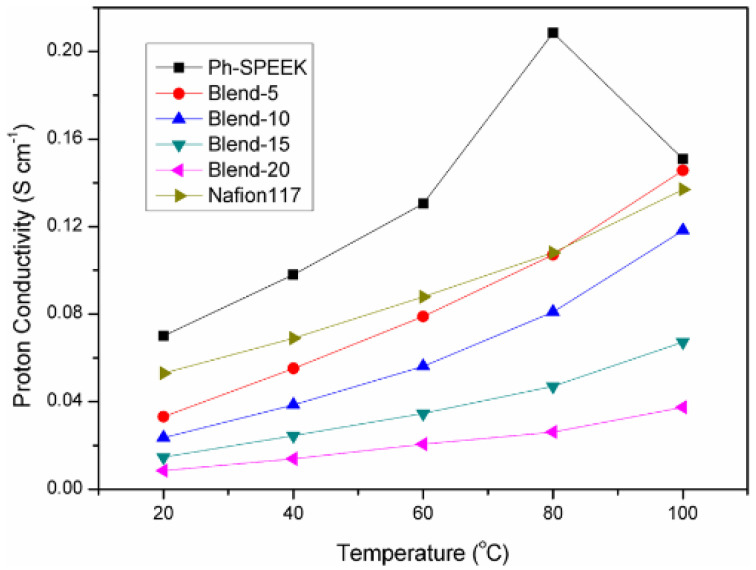
Proton conductivity of phenylated sulfonated poly(ether ether ketone), Nafion, and blend membranes [118]. Reproduced with permission from [118] Copyright 2013, SAGE Publications Ltd.

Pengju Feng [118] synthesized a phenylated polybenzimidazole (Ph−PBI) containing flexible ether bonds and lateral phenyl groups, and SPEEK with pendant phenyl groups (Ph−SPEEK) [119]. Synthesis of phenylated polybenzimidazole and Ph-SPEEK was illustrated in Figure 20 and Figure 21. The two polymers were blended by using a solution casting method to obtain the composite membrane. Due to the acid–base interactions and cross-linking between ions, the oxidative stability and mechanical properties of the blended membrane were significantly improved, and the water uptake and swelling ratio were markedly reduced. At 80 °C, the proton conductivity of the Blend-5 (with 5 wt% Ph-PBI content) composite membrane was 0.107 S cm^−1^, which is comparable to that of Nafion 117 (0.108 S cm^−1^). The proton conductivities of composite membranes were shown in Figure 22. As the Ph-PBI content increased from 5 wt% to 20 wt%, the tensile modulus of the blended membrane increased from 0.51 GPa to 0.82 GPa, and the tensile strength increased from 22.3 MPa to 37.2 MPa, with both being higher than that of the original Ph-SPEEK membrane.

From the above work, it can be seen that the addition of PBI reduced the proton conductivity of the composite membrane, but the chemical stability and mechanical properties of the composite membrane were significantly higher than those of SPEEK. To simultaneously improve the proton conductivity and mechanical properties of membranes, Shuitao Gao [120] prepared highly sulfonated poly(ether ether ketone) and highly sulfonated polybenzimidazole (SPBI), and composited the two to prepare SPEEK/SPBI blended membranes. This not only enhanced the electrochemical performance but also improved the mechanical properties.

SPBI is an amphiphilic polymer. On the one hand, PBI after sulfonation has acidity, and due to its similar structure to SPEEK, it can be miscible with SPEEK; on the other hand, PBI contains nitrogen-containing basic groups that can form acid–base interactions with the acidic -SO_3_H in SPEEK, reducing the high hydrophilicity of SPEEK and enhancing its stability. To further improve the proton conductivity of membranes, sulfonated graphene oxide (S-GO) was added as a filler to the SPEEK/SPBI composite membrane, which could compensate for the proton conductivity to some extent. Contrary to graphene, graphene oxide has electronic insulating properties, and oxidation makes GO easily hydrated, capable of regulating the state of water in the water channels to adjust the proton conductivity. Additionally, GO has a high specific surface area and itself is a good proton conductor, thus it can serve as an excellent composite filler to enhance the proton conductivity of the composite membranes.

**Figure 23:**
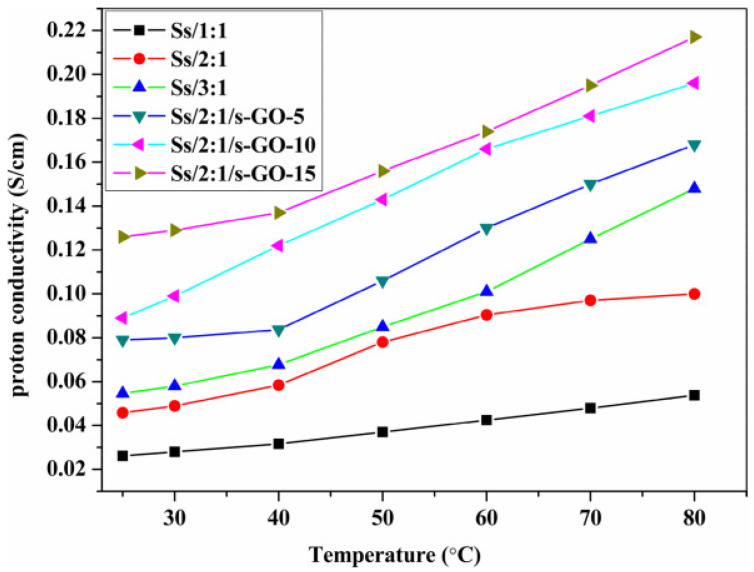
The proton conductivity of SPEEK/SPBI/S-GO composite membranes [120]. Reproduced with permission from [120] Copyright 2018, Wiley.

Figure 23 descrbed the proton conductivity of SPEEK/SPBI/S-GO composite membranes. When the content of S-GO added is below 15 wt%, the proton conductivity is enhanced due to strong interfacial interactions and the presence of hydrogen bonds. The results show that when the loading amount of S-GO is at 15 wt%, the proton conductivity of the Ss/2:1/s-GO-15 membrane (with a mass ratio of SPEEK/SPBI = 2:1 containing 15 wt% S-GO) can reach up to 0.217 S cm^−1^, which is 2.17 times that of the Ss/2:1 membrane (0.1 S cm^−1^).

The composite membrane also achieved superior chemical stability. The Ss/2:1 membrane, without the addition of S-GO fillers, had a tensile strength of 12.0 MPa at an elongation of 14.5%. Due to the presence of hydrogen bonds between S-GO and the S-PEEK/S-PBI matrix, as the content of S-GO fillers increased, strong interfacial adhesion and cross-linking structures were formed in the membrane, which further increased the tensile strength and Young’s modulus of the composite membrane. However, when the loading amount of S-GO reached up to 15 wt%, S-GO began to agglomerate, leading to a slight decrease in Young’s modulus and tensile strength.

**Figure 24:**
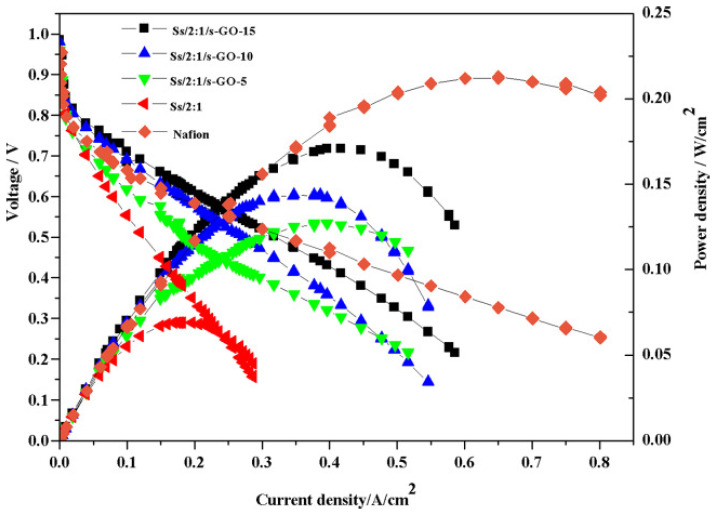
Single-cell performances of the membranes with H_2_/air operated under 25 °C. The flux rates of H_2_ and air are 100 and 150 mL/min, respectively [120]. Reproduced with permission from [120] Copyright 2018, Wiley.

In H_2_/air PEMFC single-cell tests, as shown in Figure 24, the single-cell assembled with the Ss/2:1/s-GO-15 membrane achieved a maximum power density of 171 mW cm^−2^ at a current density of 417 mA cm^−2^, which is 2.5 times that of the Ss/2:1 membrane, but is still lower than that of Nafion (211 mW cm^−2^ at 546 mA cm^−2^). High proton conductivity of the membrane is essential, but it is not sufficient for a fuel cell to have good performance. Therefore, further optimization of the Membrane Electrode Assembly (MEA) preparation conditions is necessary to enhance the overall performance of the fuel cell.

**Figure 25:**
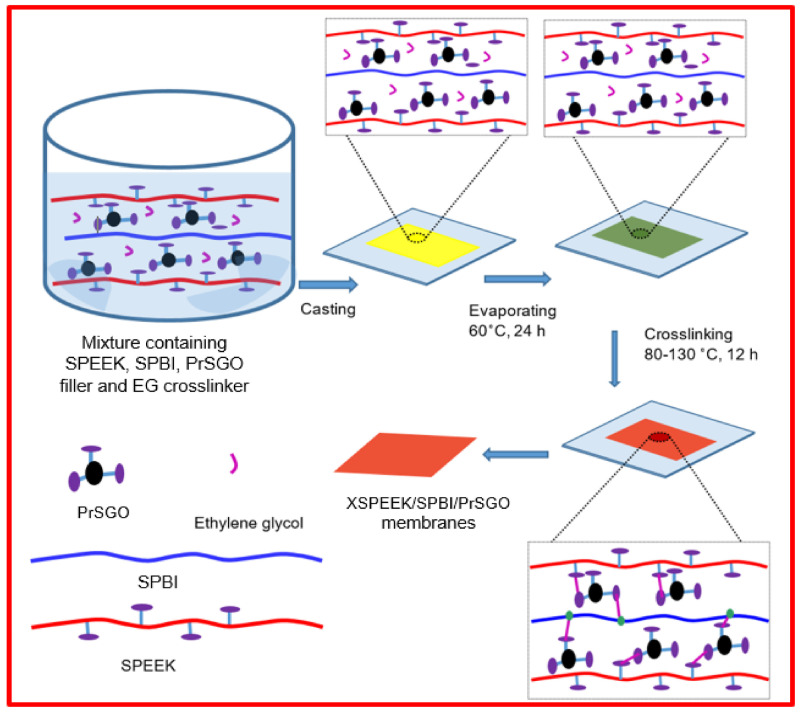
Preparation of cross-linked polymer composite membranes using SPEEK and SPBI as polymer matrix and PrSGO as filler [121]. Reproduced with permission from [121] Copyright 2023, Wiley.

Tushar Kanti Maiti [121] used a composite membrane of SPEEK and SPBI as the base membrane and then grafted propylsulfonic acid-functionalized graphene oxide (PrSGO) as a filler onto the base membrane to prepare crosslinked SPEEK/SPBI/PrSGO composite membranes via a solution casting method. The preparation is described in Figure 25. The results showed that due to the addition of SPBI, the interaction between the basic and acidic groups in the mixture increased, reducing the movement of the polymer chain segments, which significantly increased the glass transition temperature (T_g_) of the crosslinked blend membrane. Moreover, with the addition of PrSGO, hydrogen bonds and strong dipole interactions were formed between it and the SPEEK/SPBI matrix, which also helped to increase the T_g_ of the membranes. The incorporation of PrSGO also improved the mechanical properties of the composite membrane. The addition of the PrSGO filler increased the elastic modulus of the membrane from 410.56 MPa to 1056.76 MPa.

**Figure 26:**
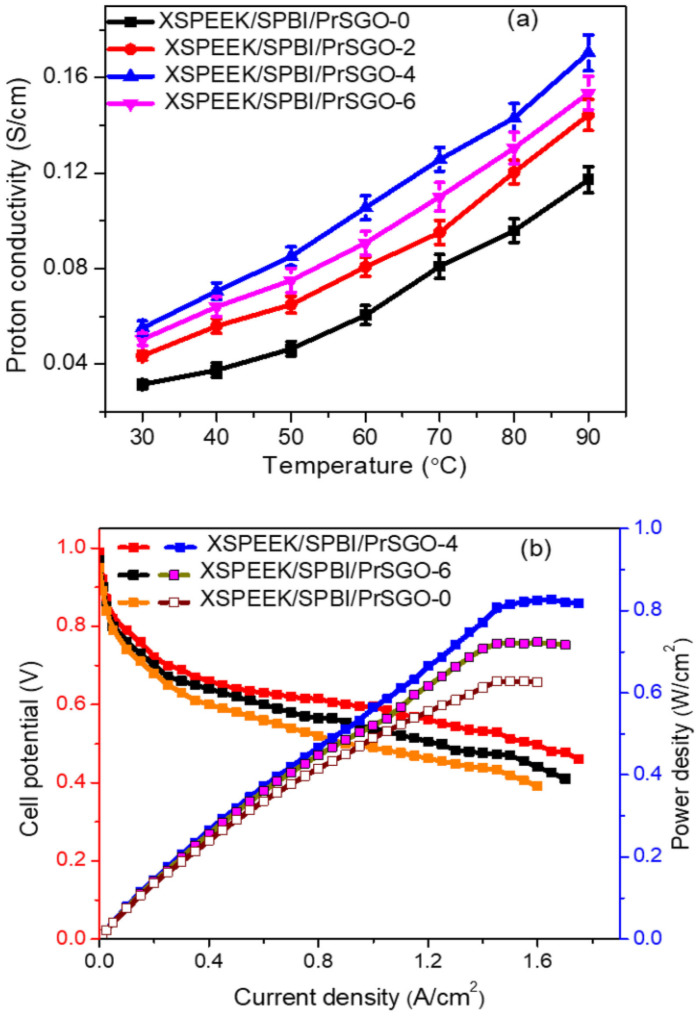
(**a**) Proton conductivity of XSPEEK/SPBI/PrSGO composite membranes, (**b**) performance of single cell with XSPEEK/SPBI/PrSGO nanocomposite membranes at 80 °C and 100% RH [121]. Reproduced with permission from [121] Copyright 2023, Elsevier Ltd.

The incorporation of PrSGO increased the content of sulfonic acid groups, enhancing the water uptake of the membrane, and creating more proton conduction sites on its surface, thereby reducing the hindrance to proton conduction in the composite membrane. Studies have shown that within a certain range, the addition of fillers enhances the proton conductivity of membranes. However, when the content of PrSGO is excessive, it may aggregate, thus reducing the proton conductivity. At 90 °C and 100% RH, when the content of PrSGO is 4 wt%, the proton conductivity of the crosslinked SPEEK/SPBI/PrSGO composite membrane (XSPEEK/SPBI/PrSGO-4) can reach up to 170 mS cm^−1^. At 80 °C and 100% RH, a fuel cell assembled with XSPEEK/SPBI/PrSGO-4 achieved a maximum power density of 0.82 W/cm^2^, showing great potential for applications. The proton conductivity of composite membranes and the performance of single cell with XSPEEK/SPBI/PrSGO nanocomposite membranes are shown in Figure 26.

Combining SPEEK (containing acidic groups) with PBI (containing alkaline groups) to form ion crosslinking is a good method, which can maintain proton conductivity and improve mechanical properties at the same time. However, in the operating environment of fuel cells, these alkaline groups are easily leached out in liquid water, resulting in a decrease in the PEMFC output performance.

**Figure 27:**
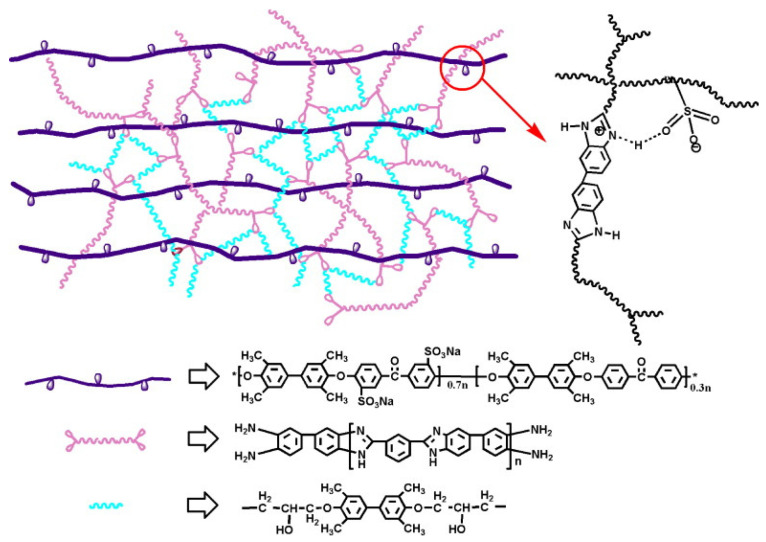
Schematic representation of the SPEEK/o-PBI/TMBP composite membranes [122]. Reproduced with permission from [122] Copyright 2011, Elsevier.

Miaomiao Han et al. [122] synthesized oligomers of polybenzimidazole terminated with diamines (o-PBI) and combined them with SPEEK and 4,4′-dihydroxybiphenyl epoxy resin (TMBP) to prepare SPEEK/o-PBI/TMBP composite membranes for DMFCs. Figure 27 shows the schematic representation of the SPEEK/o-PBI/TMBP composite membranes. The PBI oligomers and epoxy resins undergo in-situ polymerization reactions within the SPEEK, forming a semi-interpenetrating polymer network within the membrane. Due to the acid–base interactions between the sulfonic acid groups and the benzimidazole groups, as well as the crosslinking reactions between the PBI and the epoxy resin structures, a dense three-dimensional network structure is formed within the composite membrane, which effectively limits the water uptake and swelling ratio, giving the membrane good dimensional stability. Meanwhile, with the increase of the content of o-PBI/TMBP, both the tensile strength and elongation at the break of the composite membrane increase. This is because the three-dimensional network structure of the composite membrane restricts the movement of the polymer chains, thereby enhancing its mechanical properties. Test results indicate that SPEEK/o-PBI/TMBP-15 (the composite membrane containing 15 wt% o-PBI/TMBP loading) has the highest proton conductivity, reaching up to 0.142 S cm^−1^ at 80 °C, which is close to that of Nafion (0.146 S cm^−1^). The composite membrane SPEEK/o-PBI/TMBP-15 (with 20 wt% o-PBI/TMBP loading) exhibits the lowest proton conductivity (0.130 S cm^−1^). The proton conductivity of SPEEK/o-PBI/TMBP-15 is higher than other composite membranes with the same degree of hydration, suggesting that the benzimidazole groups and the crosslinking structure of o-PBI/TMBP can reduce the dependence of proton conductivity on water. Furthermore, since the N-sites on the imidazole groups can act as both proton donors and acceptors, they can capture free radicals generated during the operation of the fuel cell, mitigating the attack of free radicals and thus inhibiting membrane degradation, enhancing its oxidative stability, and extending the service life of the membrane material. The methanol permeability of the composite membrane (2.38 × 10^−8^ cm^2^ s^−1^) was one order of magnitude lower than that of SPEEK (14.63 × 10^−8^ cm^2^ s^−1^) and two orders of magnitude lower than that of Nafion (1.005 × 10^−6^ cm^2^ s^−1^). Therefore, SPEEK/o-PBI/TMBP-15 possesses excellent permselectivity.

**Figure 28:**
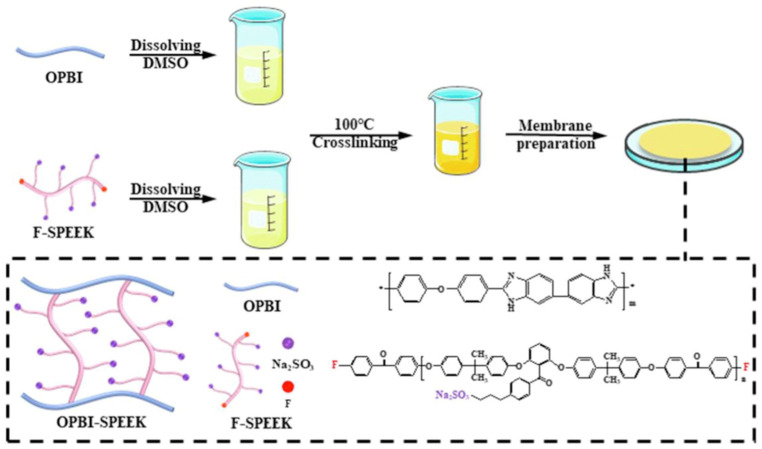
Preparation process of OPBI−SPEEK membranes [123]. Reproduced with permission from [123] Copyright 2021, Elsevier.

Improving the mechanical properties of composite membranes through acid–base reactions or N-substitution reactions of PBI can enhance their performance, but these membranes may not perform well at low temperatures. To address this issue and expand the operational temperature range of the membranes, aiming for the low-temperature startup of fuel cells, Jierui Song [123] synthesized a polymer with fluorine-terminated SPEEK (F-SPEEK) through an aromatic nucleophilic substitution reaction, giving it the functionality of a crosslinking agent. The preparation process is illustrated in Figure 28. Subsequently, a crosslinked composite membrane was prepared by adding it to poly(4,4′-biphenylene-5,5′-bibenzimidazole) (OPBI) through a typical N-substitution reaction. Since SPEEK exhibits excellent proton conductivity under hydrated conditions at low temperatures (40~80 °C) and OPBI shows good proton conductivity at high temperatures (80~160 °C), the blend membrane demonstrated excellent overall battery output performance across a wide temperature range (40–160 °C). This achievement facilitated the low-temperature startup of fuel cells and provided valuable experience for the room-temperature startup of HT-PEMFCs. Since F-SPEEK is a fluorinated composite, it does not have significant advantages in terms of environmental protection compared to non-fluorinated membranes.

**Figure 29:**
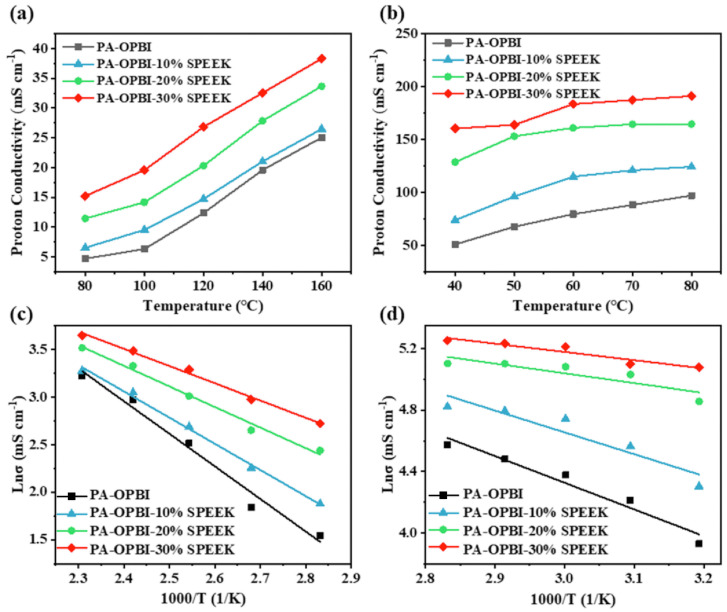
Proton conductivity for OPBI and OPBI−SPEEK membranes at high temperature from 80 to 160 °C under anhydrous: (**a**), at low temperature from 40 to 80 °C under 98% RH (**b**); proton conductivity Arrhenius plots of for OPBI and OPBI−SPEEK membranes at high temperature from 80 to 160 °C under anhydrous (**c**), at low temperature from 40 to 80 °C under 98% RH (**d**). [123]. Reproduced with permission from [123] Copyright 2021, Elsevier.

As is shown in Figure 29, under the conditions of 80 °C and 98% RH, the blend of OPBI with 30 wt% SPEEK, namely the OPBI-30 wt% SPEEK membrane, achieved a proton conductivity of 191 mS cm^−1^. In single-cell fuel cell tests, both the OPBI and the OPBI-30 wt% SPEEK membranes exhibited high open-circuit voltage values, indicating their low gas permeability. Due to its denser structure, the OPBI-30 wt% SPEEK membrane demonstrated a higher power density compared to OPBI. At 80 °C and 100% RH, the maximum power density reached 115.7 mW cm^−2^, and under anhydrous conditions at 160 °C, the maximum power density reached 193.2 mW cm^−2^.

**Figure 30:**
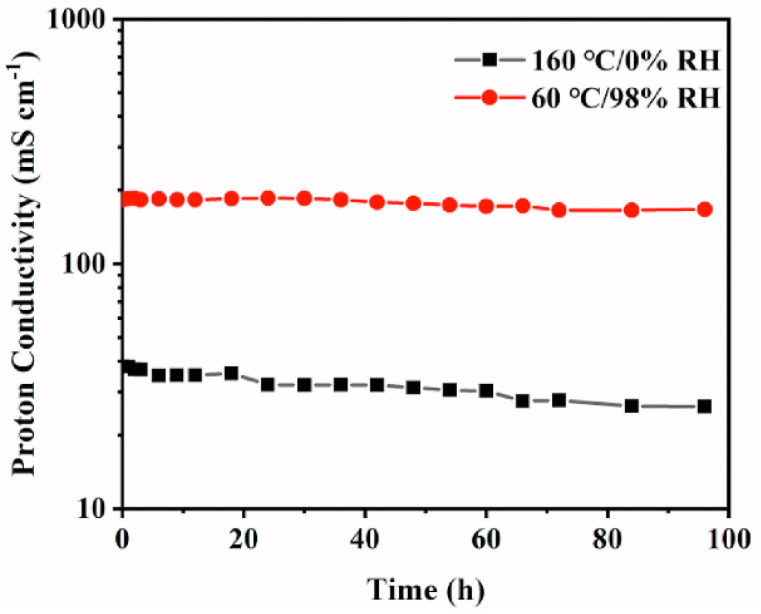
Stability test of proton conductivity for the OPBI−30% SPEEK at 160 °C under 0% RH and at 60 °C under 98% RH [123]. Reproduced with permission from [123] Copyright 2021, Elsevier.

F-SPEEK and OPBI form both hydrogen bonds and covalent bonds, which hinder the oxidation by free radicals, resulting in the SPEEK-OPBI membrane showing slightly higher chemical stability than OPBI alone after 120 h of testing in Fenton’s solution. It is also due to the formation of more hydrogen bonds and covalent bonds between the polymers that the mechanical properties of the composite membrane are significantly enhanced. The tensile strength of the OPBI-30%SPEEK membrane, which contains 30 wt% SPEEK, is 41.1 MPa, a 133% increase compared to the original OPBI membrane. The stability test of proton conductivity for the OPBI-30% SPEEK is shown in Figure 30.

The presence of both phosphate and sulfonate groups in the blended membrane provides more proton transfer sites for proton hopping, leading to better proton conductivity of the composite membrane. SPEEK promotes microphase separation in the blend membrane, facilitating the construction of hydrophilic–hydrophobic channels. The SEM images of the composite membrane also confirm the occurrence of microphase separation within the membrane.

The properties of SPEEK/Imidazole composite membranes are summarized in Table 2.

### 3.3. SPEEK/Fluorinated Polymer Composite Membrane

Polyvinylidene fluoride (PVDF) is a hydrophobic polymer with a regular molecular chain structure. The fluorine atoms (0.071 nm) and hydrogen atoms (0.037 nm) have small radii, resulting in a strong hydrogen bonding effect, which endows the polymer with high strength and good toughness. Due to the high bond energy of the C-F bond, PVDF exhibits excellent mechanical properties and good chemical stability, and its outstanding performance is gaining an increasing amount of attention. PVDF can generate stronger intermolecular interactions with SPEEK of a lower DS, thereby demonstrating better miscibility. Improved miscibility is also beneficial to the transport properties of the membrane, providing a more continuous proton channel.

Generally, due to the lack of proton conductivity for PVDF, blending SPEEK with PVDF reduces proton conductivity by decreasing the molar concentration of sulfonic acid groups and dispersing proton transport sites, leading to a decline in the proton conductivity of the blended membranes. However, the hydrophobicity of PVDF impedes the permeation of methanol through the membrane, enhancing its methanol resistance. Consequently, SPEEK/PVDF composite membranes are extensively employed in DMFCs [124], and incorporating a small amount of the hydrophobic polymer PVDF into SPEEK has proven effective in achieving both dimensional stability and an optimal DMFC output performance. The membrane selectivity is typically defined as the ratio of proton conductivity to methanol permeability, and it is considered a key parameter for determining the optimal output performance of DMFCs and a primary indicator of the overall performance for a membrane in DMFCs.

**Figure 31:**
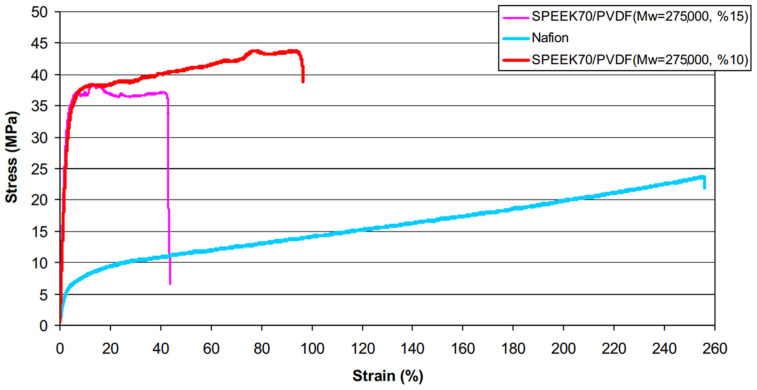
The stress–strain plots of SPEEK/PVDF blended membranes [125]. Reproduced with permission from [125] Copyright 2010, Elsevier.

Inan [125] blended SPEEK (DS = 70%) with PVDF of three different molecular weights to prepare blended membranes, where the molecular weights of PVDF were: 18,000/275,000/530,000. As expected, the blended membranes with added PVDF all exhibited lower water uptake and proton conductivity compared to original SPEEK, and higher oxidative and mechanical stability than SPEEK. The study found that when the mass fraction of PVDF with a molecular weight of 530,000 was 10 wt%, the SPEEK/PVDF blended membrane exhibited the highest proton conductivity, reaching 145.4 S cm^−1^. The blended membrane of PVDF with Mw of 275,000 and SPEEK showed a proton conductivity of 123.6 S cm^−1^, and upon calculation, it had the smallest standard deviation, indicating better miscibility. The SPEEK70/PVDF (M_w_ = 180,000) and SPEEK70/PVDF-HFP blended membranes completely degraded within 2 to 3 h, while the SPEEK70/PVDF (M_w_ = 275,000) and SPEEK70/PVDF (M_w_ = 530,000) blended membranes completely degraded within 3 to 4 h. In terms of methanol permeability, the SPEEK70/PVDF (M_w_ = 275,000) blend membrane also showed the lowest methanol permeability value (3.13 × 10^−7^ cm^2^/s), which is significantly lower than that of Nafion 117 (1.21 × 10^−6^ cm^2^/s). Additionally, in permeability tests for hydrogen and oxygen, the hydrogen permeability was one-tenth of that of Nafion, and the oxygen permeability was one-fifth of that of Nafion. The blend membranes containing PVDF all exhibited higher tensile stress than Nafion but showed a lower elongation at break. With the increase of PVDF content, the elongation at break of the SPEEK70/PVDF (M_w_ = 275,000) composite membrane was lower after 10 wt% PVDF, while the high content of PVDF had no effect on the Young’s modulus. The stress–strain plots of SPEEK/PVDF blended membranes are shown in Figure 31. These results indicate that PVDF with a molecular weight of 275,000 is likely to be a promising choice for SPEEK/PVDF blended membranes.

Researchers also blended SPEEK (DS = 70%) with 5–20 wt% polyvinylidene fluoride-hexafluoropropylene (PVDF-HFP) with a molecular weight of 130,000 to prepare blended membranes via the solution casting method. In the SEM results, phase separation in the membrane structure was observed and it can be concluded that PVDF-HFP and SPEEK are thermodynamically immiscible due to their structural differences. Therefore, PVDF-HFP must form dispersed or co-continuous phases to be used as a PEM. This could be due to the density differences between the separated PVDF-HFP-rich and SPEEK-rich liquid domains, and it may also be related to the variation of solvent concentration from the surface to the interior. The immiscibility of the two was also confirmed in the glass transition temperature tests. For the SPEEK/PVDF-HFP membranes (PVDF-HFP, Mn = 130,000), a glass transition was observed at 185 °C, and a melting point was observed at 146 °C. They consider that the SPEEK/PVDF-HFP blend membranes at this ratio are not suitable for use in PEMFCs.

Daud et al. [126] blended highly sulfonated SPEEK (DS = 80%) with PVDF. SEM results indicated that the SPEEK/PVDF15 blend membrane, containing 15% weight fraction of PVDF, exhibited better compatibility and uniformity in composition compared to other membranes in the same series. Due to the poor proton conductivity of PVDF, which was approximately 0.044~0.113 mS cm^−1^, the incorporation of PVDF compromised the proton conductivity of SPEEK. Results showed that the SPEEK/PVDF15 had the highest proton conductivity, reaching up to 46.23 mS cm^−1^ at 80 °C (about 17.2% lower than Nafion 117). The addition of PVDF significantly reduced the degree of swelling in the blended membrane. In the water uptake tests, it was observed that the water uptake first increased and then decreased with the increase in PVDF content. The SPEEK/PVDF10 and SPEEK/PVDF15 membranes had a finer and more uniform morphological structure, with surfaces conducive to water uptake and ion transport, exhibiting water uptake rates of 63.80% and 63.99% at room temperature, respectively. In contrast, SPEEK/PVDF20, due to the lower uniformity of the blended polymers, reduced the sorption capacity for water molecules and the migration rate of ions, resulting in a very low water uptake rate of 46.95% at room temperature. Test results indicated that as the content of PVDF increased, the tensile strength of the blend membrane showed an upward trend, demonstrating that PVDF significantly enhanced the mechanical properties of the membrane.

**Figure 32:**
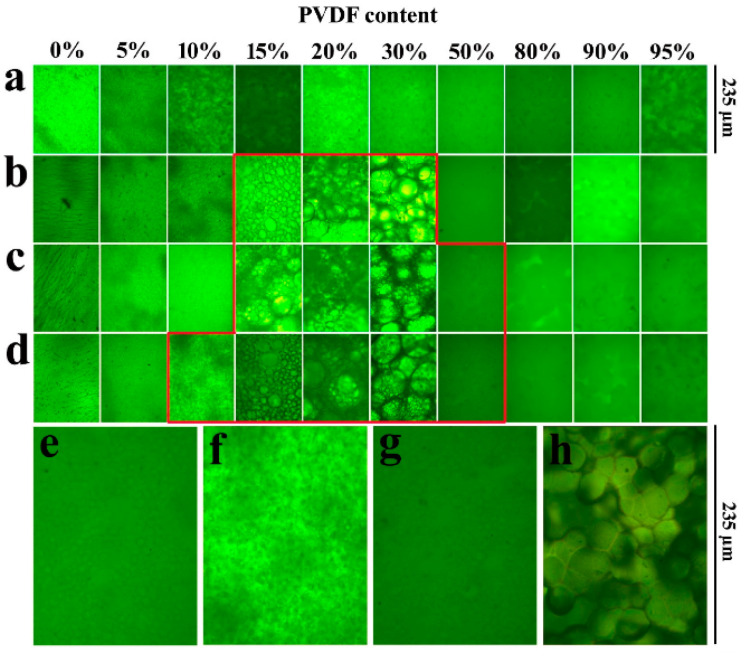
Optical micrographs of (**a**) SPEEK44/PVDF, (**b**) SPEEK56/PVDF, (**c**) SPEEK67/PVDF, and (**d**) SPEEK73/PVDF blended membranes with different PVDF contents, (**e**) SPEEK67/PVDF-50, (**f**) SPEEK73/PVDF-10, (**g**) SPEEK73/PVDF-50 blended membranes, and (**h**) PVDF [127]. Reproduced under terms of the CC-BY license [127]. Copyright 2019, *MDPI*.

He et al. [127] blended SPEEK of varying DS with PVDF to prepare composite membranes and studied their transport properties. Optical microscopy results confirmed that SPEEK with a lower degree of sulfonation had better compatibility with PVDF and reduced the crystallinity of PVDF. As shown in the Figure 32, The blend membrane within the red solid line area exhibits significant phase separation the SPEEK44/PVDF blend membrane with a DS of 44% exhibited no signs of phase separation across the entire range of PVDF content.

In DMFCs, the presence of PVDF significantly reduced the fuel permeability, and both proton conductivity and methanol permeability decreased with the increase of PVDF content. The research conducted by He and colleagues indicated that SPEEK/PVDF membranes with better miscibility have more promising applications in DMFCs.

Studies have indicated that due to its high proton conductivity and water retention capability, the addition of phosphoric acid boron (BPO_4_) can enhance the proton conductivity of SPEEK [128]. Aygün Çalı et al. [129] developed the SPEEK/PVDF blend membrane with the addition of phosphoric acid boron, which exhibited better water retention, ion exchange capacity (IEC), and proton conductivity compared to original Nafion and SPEEK membranes. When the content of phosphoric acid boron (BPO_4_) was increased from 2 wt% to 15 wt%, the IEC of the SPEEK/PVDF blended membrane increased from 0.872 meq g^−1^ to 1.64 meq g^−1^, which was significantly higher than that of Nafion 117 (0.9 meq g^−1^).

To improve the reduced proton conductivity caused by the introduction of PVDF, polyvinylidene fluoride co-hexafluoropropylene copolymer (PVDF-co-HFP) was used to replace or compensate for the hydrophobicity of PVDF. PVDF-co-HFP is a block polymer that contains both hydrophilic sulfonic acid groups and vinylidene fluoride blocks. The presence of sulfonic acid groups enhances proton conduction sites and provides additional proton conduction pathways. The excellent mechanical stability and acceptable proton conductivity make composite membranes containing PVDF-co-HFP highly advantageous in fuel cell applications [130,131].

Ahmad Bagheri [132] prepared SPEEK (DS = 68%) and PVDF-co-HFP, as well as sulfonated PVDF-co-HFP (SPVDF-co-HFP) copolymers (DS = 31%). Composite membranes were prepared by blending different proportions of PVDF-co-HFP and SPVDF-co-HFP with SPEEK using the solution casting method. The test results indicate that with the increase of PVDF content in the composite membrane, hydrogen bonds are formed between the SO_3_H groups of SPEEK and SPVDF-co-HFP polymers. This helps prevent excessive expansion, reducing the water uptake rate of the composite membrane and improving its stability, while also reducing methanol fuel permeability. The hydrogen bonding in the blend simultaneously reduces the content of hydrolyzable sulfonic acid groups in the polymer structure, leading to a decrease in proton transport sites, which in turn results in a decrease in the IEC values of both blended membranes. Among them, the IEC value of SPEEK membrane doped with PVDF-co-HFP decreased from 1.61 meq g^−1^ to 0.92 meq g^−1^. The IEC value of SPEEK blended membranes doped with SPVDF-co-HFP decreased from 1.61 meq g^−1^ to 1.28 meq g^−1^.

**Figure 33:**
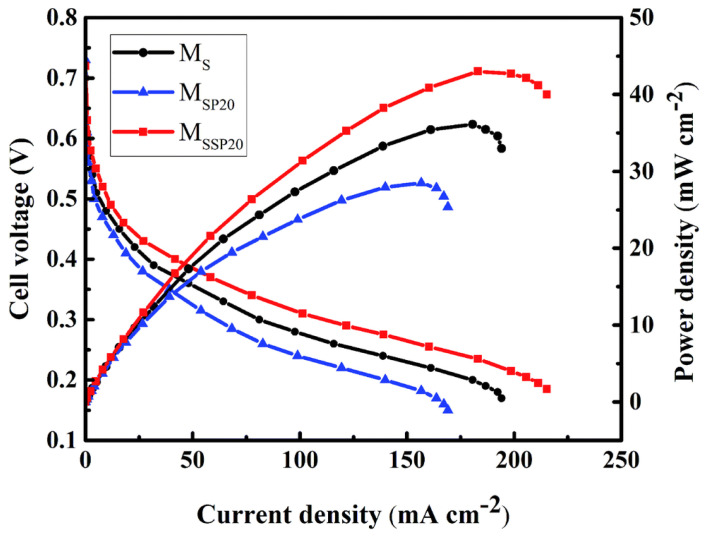
Current density−potential (I–V) and power density curves of the DMFC assembled with different membranes at 30 °C [132]. Reproduced with permission from [132] Copyright 2016, Royal Society of Chemistry.

Since PVDF does not possess proton conductive capabilities, the proton conductivity of the composite membrane is lower than that of original SPEEK ones. However, SPVDF-co-HFP contains hydrophilic sulfonic acid groups, hence why the blended membrane with SPEEK exhibits better proton conductivity compared to the blend of PVDF-co-HFP with SPEEK. The proton conductivity results in the following order: SPEEK > SPEEK/SPVDF-co-HFP > SPEEK/PVDF. The study indicates that the blended membrane containing SPEEK/SPVDF-co-HFP (10 wt%) reaches the highest proton conductivity at 20°C, which is 37.03 mS cm^−1^. This is slightly lower than that of SPEEK (42 mS cm^−1^). The composite membranes with 20 wt% PVDF-co-HFP and 20 wt% SPVDF-co-HFP exhibit the highest permselectivity. In detail, the permselectivity of SPEEK/PVDF-co-HFP (20 wt%) is 7.27 × 10^4^ S^2^ cm^−3^, which is slightly lower than the permselectivity of SPEEK membrane (7.37 × 10^4^ S^2^ cm^−3^). The permselectivity of SPEEK/SPDF-co-HFP (20 wt%) was the highest, reaching up to 15.51 × 10^4^ S^2^ cm^−3^. This also endowed it with the best cell performance. At 30°C, SPEEK/SPDF-co-HFP (20 wt%) achieved a maximum power density of 43.02 mW cm^−2^ at a current density of 215.3 mA cm^−2^, which is higher than SPEEK (with a maximum power density of 36.12 mW cm^−2^ at 194.1 mA cm^−2^). The current density−potential (I–V) and power density curves of the DMFC assembled with different membranes are shown in the Figure 33.

Researchers are also committed to finding other materials to composite on this basis, in order to improve the comprehensive performance of the composite membrane. Silica (SiO_2_) is an important inorganic filler commonly used in fuel cells and has played a significant role in enhancing the function of fuel cells. Currently, SiO_2_ has been widely applied in various base membranes, such as perfluorinated membranes (Nafion) and sulfonated membranes like SPEEK, SPBI, SPI, etc.

P. Martina [133] developed composite membranes of SPEEK/SPVdF-HFP doped with sulfonated silica (S-SiO_2_). The nano S-SiO_2_, which had a high specific surface area, not only increased the concentration of -SO_3_H but also formed strong acid–acid interactions with the SPEEK/SPVdF-HFP matrix, promoting the formation of hydrophilic channels and significantly enhancing the proton conductivity of the composite membrane. The proton conductivity of SPEEK/SPVdF-HFP/S-SiO_2_ (6 wt%) at 90 °C could reach 79 mS cm^−1^, which was higher than that of SPEEK. Moreover, the hydrogen bonds formed between the -SO_3_H groups in S-SiO_2_ and the -OH groups increased the tensile strength and elastic modulus of the composite membrane. Single-cell test results at 90 °C and 100% RH showed that the current density of SPEEK/SPVdF-HFP/S-SiO_2_ (6 wt%) could reach 354 mA cm^−2^, with a power density of 110 mW cm^−2^, indicating that the composite membrane had good durability.

**Figure 34:**
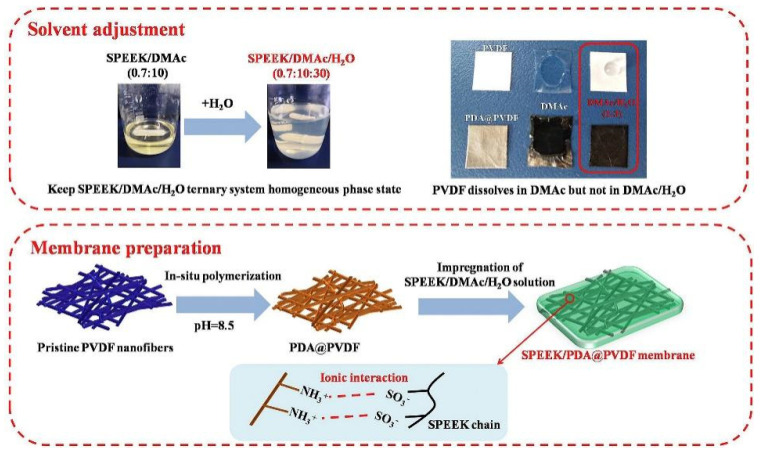
Schematic illustration of the synthesis process and microstructure of SPEEK/PDA@PVDF composite membrane [134]. Reproduced with permission from [134] Copyright 2019, Elsevier.

Guoliang Liu et al. [134] fabricated a nanofiber composite membrane of SPEEK and PVDF for DMFC via electrospinning. The synthesis process and microstructure of SPEEK/PDA@PVDF composite membrane were shown in Figure 34. Initially, SPEEK (DS = 76.7%) was prepared, followed by the creation of PVDF nanofiber mats through electrospinning. The mats were then surface-modified using polydopamine (PDA) to obtain PDA-modified PVDF (PDA@PVDF) nanofiber mats. Owing to the strong adhesion and hydrophilic nature of PDA, the presence of PDA improved the interfacial bonding between SPEEK and PVDF, endowing the composite membrane with hydrophilicity and enhanced toughness. Finally, the SPEEK-filled nanofiber membrane was prepared using a dip-coating method, resulting in the SPEEK/PDA@PVDF composite membrane. To prevent the solvent in SPEEK from disrupting the PVDF fiber skeleton, a ternary system of SPEEK/solvent/non-solvent was employed.

PVDF possessed strong hydrophobicity, whereas PDA@PVDF nanofiber mats exhibited hydrophilicity. This characteristic could partially compensate for the reduction in water uptake and proton conductivity caused by the blending of SPEEK and PVDF. It promoted the formation of a hydrogen bond network in an aqueous environment, which in turn facilitated the diffusion of protons along the network. The acid–base pairs formed by -SO_3_^2−^ and -NH_3_^+^ could lower the barrier for proton conduction, thereby increasing the proton transfer rate. As expected, the incorporation of inert PVDF sacrificed the proton conductivity of the membrane. The proton conductivity of SPEEK at 80 °C was 0.11 S cm^−1^, while the proton conductivity of SPEEK/PVDF was 0.056 S cm^−1^. Fortunately, by improving the hydrophilicity of PVDF through PDA, the SPEEK/PDA@PVDF membrane exhibited a proton conductivity of 0.06 S cm^−1^ under the same conditions.

SPEEK is rigid with a fracture elongation of just 35%. With the addition of PDA@PVDF, the interfacial interactions between the components were enhanced, leading to a more compact membrane structure and significantly improved mechanical properties. The elongation at break of the SPEEK/PVDF and SPEEK/PDA@PVDF blended membranes were 143% and 188%, respectively, demonstrating that they exhibit superior flexibility compared to original SPEEK ones.

**Figure 35:**
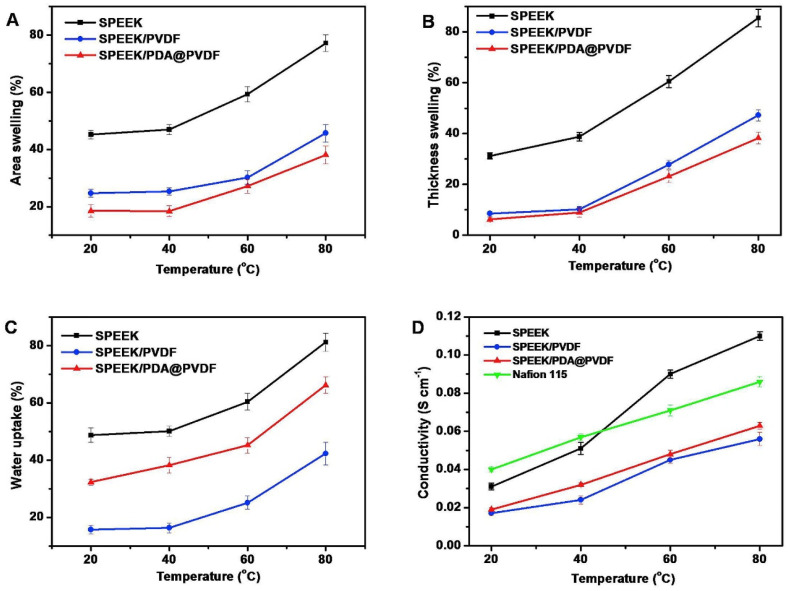
(**A**) Area swelling, (**B**) thickness swelling, (**C**) water uptake, and (**D**) proton conductivity of SPEEK and composite membranes at different temperatures [134]. Reproduced with permission from [134] Copyright 2019, Elsevier.

In DMFCs, methanol permeability is a key factor influencing the output performance of DMFCs. If the methanol permeability is excessively high, the movement of methanol from the anode to the cathode can impede the reaction between oxygen and protons, reduce the open-circuit voltage, and ultimately lower the efficiency and performance of fuel cells. In the SPEEK/PDA@PVDF composite membrane, the presence of the PVDF fiber skeleton forms a dense structure that complicates the pathways of methanol. The movement of methanol is constrained by the fiber skeleton, thereby imparting the composite membrane with superior methanol-blocking capabilities. At 70 °C, Nafion 115 exhibits a methanol permeability of 79.2 × 10^−7^ cm^2^ s^−1^, while the SPEEK membrane displays a methanol crossover rate of 48.3 × 10^−7^ cm^2^ s^−1^. In contrast, the SPEEK/PDA@PVDF composite membrane shows a methanol permeability of only 3.8 × 10^−7^ cm^2^ s^−1^ at 70 °C. In single-cell tests, the maximum power density of the SPEEK/PDA@PVDF membrane is 92.0 mW cm^−2^ and 104.0 mW cm^−2^ at methanol concentrations of 2 M and 5 M, respectively, surpassing 89.3 mW cm^−2^ and 84.0 mW cm^−2^ of commercial Nafion 115 under the same conditions. Figure 35 shows area swelling, thickness swelling, water uptake, and proton conductivity of SPEEK and composite membranes.

**Figure 36:**
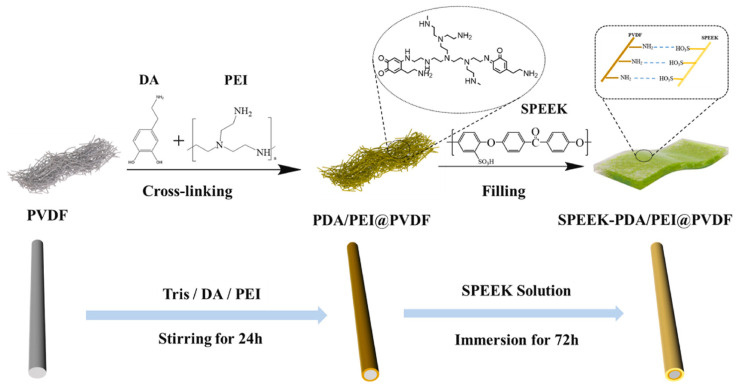
Preparation process of SPEEK-PDA/PEI@PVDF composite membranes [135]. Reproduced with permission from [135] Copyright 2024, Elsevier.

Jiale Chu [135] modified PVDF nanofibers with poly-dopamine/polyethyleneimine (PDA/PEI) via a co-deposition method to obtain modified fibers with super-hydrophilicity, and then filled SPEEK into the nanofibers to prepare SPEEK-PDA/PEI@PVDF composite membranes for DMFC applications. Preparation process is shown in Figure 36. The modified PVDF has good compatibility with SPEEK, and strong interactions between -NH_2_ and -SO_3_H significantly improved the toughness of the composite membrane, with a tensile strength of 34 MPa and an elongation at break of up to 200%. Additionally, PVDF had a strong supporting effect. The strong interactions between SPEEK and PDA/PEI@PVDF allowed SPEEK to adhere firmly to the PVDF substrate, thereby limiting the swelling of SPEEK and enhancing the dimensional stability of the composite membranes. Due to the presence of -SO_3_H and its acid–base interaction with -NH_2_ of PDA/PEI, more proton conduction sites were provided, resulting in sufficient proton conductivity in the composite membranes. At 80 °C, the proton conductivity of the composite membrane could reach 48 mS cm^−1^.

The three-dimensional network structure of the inert PVDF fibers, coupled with the strong interfacial interactions between SPEEK and PDA/PEI@PVDF, significantly enhances the methanol resistance of the composite membranes. The methanol permeability of the Nafion 115 at 60 °C is 25.34 × 10^−7^ cm^2^ s^−1^, whereas the SPEEK-PDA/PEI@PVDF composite membrane exhibits a value of only 11.94 × 10^−7^ cm^2^ s^−1^ at the same temperature. Thanks to its excellent methanol resistance, during single-cell tests, the SPEEK-PDA/PEI@PVDF composite membranes demonstrated the highest power density under 2 M methanol and reached 58.9 mW cm^−2^, which is higher than that of SPEEK (47 mW cm^−2^) and Nafion 115 (48.4 mW cm^−2^) under the same conditions.

**Figure 37:**
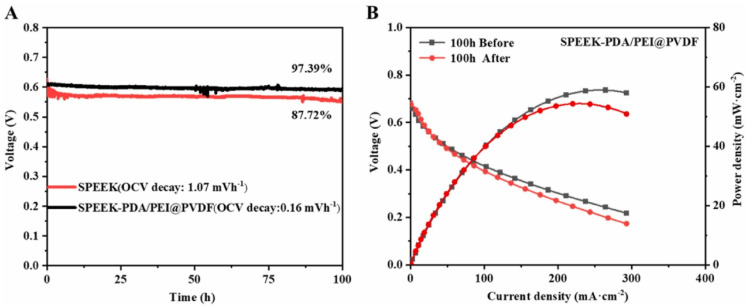
OCV curves: (**A**) and power density curves (**B**) of membranes with 2 M methanol at 80 °C [135]. Reproduced with permission from [135] Copyright 2024, Elsevier.

To measure the durability of membranes in the cell, after being tested for 100 h in the cell at 80 °C, the initial open-circuit voltage (OCV) of the SPEEK membrane was only maintained at 87.72%, whereas the OCV of the SPEEK-PDA/PEI@PVDF composite membrane remained essentially stable at 97.39% of its initial value. Moreover, after the durability test, the proton conductivity of the tested SPEEK-PDA/PEI@PVDF membrane was re-measured and compared. Its maximum power density was still as high as 54.3 mW cm^−2^, which is only a 7.8% decrease compared to the value before the durability test, whereas the OCV of SPEEK was only 87.72% of its initial value. The modified PVDF composite membrane exhibits exceptional operational stability and holds great potential for practical application in DMFCs. All the OCV curves are shown in Figure 37.

The properties of SPEEK/Fluorinated polymer composite membranes are summarized in Table 3.

The environmental concerns associated with fluorinated polymers like PVDF are significant. The production, use, and disposal of PVDF can lead to the release of per- and polyfluoroalkyl substances (PFAS), which are persistent in the environment and can accumulate in ecosystems and wildlife, posing risks to both the environment and human health. Moreover, the disposal of PVDF waste presents challenges due to its high stability, which makes it difficult to break down in the environment. While PVDF offers exceptional material properties that are valuable in many applications, it is crucial to address the environmental challenges associated with its lifecycle. This includes not only the development of more sustainable production methods but also improved recycling technologies and waste management strategies to mitigate the environmental impact of PVDF waste.

## 4. Conclusions and Outlook

This paper reviews the research progress on sulfonated poly(ether ether ketone) (SPEEK) and its composite membranes in proton exchange membrane fuel cells (PEMFCs). SPEEK is considered a promising material to replace traditional perfluorosulfonic acid membranes, such as Nafion, due to its excellent thermal stability, good mechanical properties, and tunable proton conductivity. By adjusting the degree of sulfonation (DS) of SPEEK, the hydrophilicity and proton conductivity of the membrane can be effectively controlled, while also balancing its mechanical, thermal, and chemical stability. Researchers have developed various composite membranes by combining SPEEK with a range of organic and inorganic materials, such as sulfone polymers, polybenzimidazole (PBI), fluoropolymers, and silica. These efforts aim to enhance the mechanical, chemical, and thermal stability of the membranes while reducing fuel permeability and improving the overall performance of the fuel cell. These composite membranes have shown significant advantages in improving proton conductivity, reducing methanol permeability, and enhancing durability.

Despite the great potential of SPEEK and its composite membranes in PEMFCs, there are still challenges and room for improvement. The specific points are summarized as follows:

1. The proton conductivity of the composite membrane material needs to be balanced: Reducing its water uptake and swelling to maintain the dimensional stability of the membrane often comes at the expense of reducing proton conductivity. Therefore, it is necessary to introduce materials such as graphene, functionalized graphene, or conductive inorganic materials to maintain a high level of proton conduction performance for the SPEEK membranes;
2. Chemical stability still needs to be improved: The development of new composite membranes or modification methods have effectively enhanced the antioxidant properties and chemical degradation resistance of SPEEK membranes, which is usually tested and verified using the Fenton test. However, there is still a lack of long-term in situ PEMFC operation tests, especially the dry–wet cycles during the start–stop process of the fuel cell, which may intensify the free radical reactions of the composite membrane, thereby accelerating the chemical degradation of the membrane. Current comprehensive performance tests of fuel cells lack in situ tests of the membrane to evaluate the stability of the membrane material in actual fuel cell work;
3. Cost-effectiveness still needs to be comprehensively considered based on cost–performance: Research and development of cost-effective preparation and sulfonation methods for SPEEK, as well as manufacturing processes for composite membranes, are needed to promote its commercial application. Among the currently developed composite membranes, Nafion has the highest comprehensive energy efficiency, but its high price and complex preparation process limit its widespread commercial application. In addition, Nafion membrane may degrade during long-term use. The degradation products of Nafion may include short-chain fluorides, sulfur-containing compounds, and volatile organic compounds containing fluorine. These substances may have an impact on the environment, especially when they are released into it. Fluorides and certain fluorinated compounds have been proven to be bioaccumulative and toxic, potentially posing a threat to ecosystems and human health. However, new materials that do not contain F such as SPEEK, SPBI, PVDF, SPI, etc., have been developed as a new generation of PEMs due to their price advantage and excellent mechanical properties. However, these new materials still have a significant gap compared to Nafion in terms of proton conductivity and hydrophilicity, and it is hoped that by combining them with other organic or inorganic materials with good proton conductivity and price advantage, the performance of the membrane can reach and even surpass that of Nafion, while improving the cost-effectiveness of the membrane;
4. Environmental impact assessment: It is still necessary to assess the life cycle environmental impact of SPEEK and its composite membranes in PEMFCs, especially the main components after the degradation of SPEEK, as well as whether the components after degradation are toxic, environmentally friendly, and whether they pollute the environment, which still needs to be studied, in order to truly promote the green and healthy development of sustainable energy technology;
5. Exploration of new composite strategies: Explore and develop new organic–inorganic composite strategies, using nanotechnology and other advanced materials science methods to further improve the performance of SPEEK-based membranes. For example, zipper membranes, amphoteric membranes, self-healing membranes, etc., have been reported in some applications. It is well known that PEMs are prone to stress concentration during operation, which may lead to local physical defects in the membrane material, such as pinholes, cracks, etc. Membranes with self-healing functions can recover their integrity after physical defects by the movement of molecular chain segments and the recombination of intermolecular chemical bonds. Whether such membranes can be further used in PEMFCs still needs to be explored.

## Figures and Tables

**Table 1:** The Summary of Properties of SPEEK/PSF Composite Membranes.

Membrane	Proton Conductivity (S cm^−1^)	IEC (meq/g)	WU (%)	Swelling Ratio (%)	Max Fuel Cell Power Density (mW.cm^−2^)	Max Current Density (mA cm^−2^)	Tensile Strength (MPa)	Tensile Modulus (Gpa)	Elongation at Break (%)	Methanol Penetration (cm^2^ s^−1^)	Oxidative Stability (wt%/h)	Reference
SPEEK/PSf-NBIm (2.5 wt%)	0.092 (65 °C, 100%RH)	1.26	41.6 (65 °C)	-	56	-	-	-	-	-	-	[91]
SPEEK/ PSf-Btraz (5 wt%)	0.054 (25 °C, 100%RH)	1.27 (25 °C, 100%RH)	27.2 (65 °C)	-	174	-	-	-	-	--	-	[92]
SPEEK/ PES-5	0.007	-	48	-	99.29 (60 °C, 100%RH)	367.46 (60 °C, 100% RH)	24.53	-	6.42	-	-	[94]
t-SPEEK/ SPAES (1:2:2)	0.133 (80 °C, 100%RH)	1.7	57.8	13.9	665 (80 °C, 100% RH)	-	46.5	1.5	99.1	-	0.77	[84]
CMB4	0.219	1.51	121	21.3	530.5	-	-	-	-	-	-	[95]
SPEEK/ QNPAES (6 wt%)	0.03 (60 °C, 20%RH)	2.1	43.7	42.4	150 (60 °C, 20%RH)	150 (60 °C, 20% RH)	44.2	2.4	2.4	-		[96]

**Table 2:** Summary of Properties of SPEEK/Imidazole Composite Membranes.

Membrane	Proton Conductivity (S cm^−1^)	IEC (meq/g)	WU (%)	Swelling Ratio (%)	Max Fuel Cell Power Density (mW·cm^−2^)	Max Current Density (mA cm^−2^)	Tensile Strength (MPa)		Tensile Modulus (Gpa)		Elongation at Break (%)		Methanol Penetration (cm^2^ s^−1^)	Oxidative Stability (wt%/h)	Reference
							Dry	Wet	Dry	Wet	Dry	Wet			
SPEEK/PBI	0.0046	1.58	28%	-	-	-	-		-		-		-	-	[110]
SPEEK/PBI (5 wt%)	0.080 (80 °C, 100%RH)	1.54	52.26% (80 °C)	-	-	-	47.42		1.2		15.44		5.0 × 10^−7^	-	[95]
SPEEK/ PBI (20 wt%)	0.1985 (170 °C, 100% RH) 0.099 (170 °C, 50% RH)		14.9% (30 °C)	3.1 (30 °C)	-	-	45.06		-		-		3.96 × 10^−8^	0.417	[112]
SPEEK/PBI/ BPO_4_ (20 wt%)	0.0059		16 wt%	-	-	-	-		-		-		-	-	[116]
SPEEK/ PEEK-alt-BI (15 wt%)	0.087 (80 °C, 100%RH)	1.62	130%	45	-	-	32.8		0.726		17.9		4.6 × 10^−7^	5 (began to break)	[117]
Ph-SPEEK/ Ph-PBI (5 wt%)	0.107 (80 °C, 100%RH)		21.37 wt%	55	-	-	22.3		0.51		4.05		5.27 × 10^−7^	16	[118]
Ss/2:1/s-GO-15	0.217	1.43	143.6%	63.5	171(25 °C)	417 (25 °C)	10.7		0.397		8.4		-	2.5	[120]
XSPEEK/ SPBI/PrSGO (4 wt%)	0.17 (90 °C, 100%RH)	1.94	-	-	820 (80 °C, 100%RH)	-	-				-		-	-	[121]
15 wt% oPBI/TMBP/SPEEK	0.142(80 °C)	1.32	27.62 (80 °C)	7.67 (80 °C)	-	-	52.8	22.57	1.40	0.58	21.24	14.34	2.38 × 10^−8^	0.88	[122]
OPBI- 30 wt% SPEEK	0.191 (80 °C, 98% RH)	-	-	-	115.7 (80 °C, 98%)	-	41.1		-		-		-	-	[123]
	0.038 (160 °C, 0 RH)	--		-	193.2 (160 °C, 0 RH)	-	-	-	-		-		-		

**Table 3:** Summary of Properties of SPEEK/Fluorinated polymer Composite Membranes.

Membrane	Proton Conductivity (S cm^−1^)	IEC (meq/g)	WU	Swelling Ratio (%)	Max Fuel Cell Power Density (mW·cm^−2^)	Max Current Density (mA cm^−2^)	Tensile Strength (MPa)		Tensile Stress (MPa)	Tensile Modulus (MPa)		Elongation at Break (%)		Methanol Penetration (cm^2^ s^−1^)	Oxidative Stability	Reference
							Dry	Wet		Dry	Wet	Dry	Wet			
SPEEK70/PVDF (Mw = 275,000)	0.123	-	43.8 wt%	-	-	-	34.5		-	-		70.2		3.13 × 10^−7^	3 h	[125]
SPEEK/PVDF15	0.046		130.84% (60 °C)	25(60 °C)	-	-	32.73		-	-				-	-	[126]
SPEEK/PVDF/ BP-10	0.039 (80 °C)	1.38	52%	4.8	242	400	-		36.15	-		74		-	90 h	[129]
M_SSP20_	0.032	1.35	56%	35	43.02	215.3	25.63		-	825		10.95		2.11 × 10^−7^	3 h	[132]
SPEEK/ SPVdF-HFP/ S-SiO_2_ (6 wt%)	0.079 (90 °C)	1.70	36.5%	15.9%	110	354	38.5		-	875		35.8		-	-	[133]
SPEEK/ PDA@PVDF	0.06	-	32.3%	-	104	156.5	-		-	1002	539	188	268	23.5 × 10^−7^	-	[134]
SPEEK-PDA/PEI@PVDF	0.048 (80 °C)	-	32.53% (60 °C)	19.96 (60 °C)	58.9 (80 °C)		34		-	-		174		11.94 × 10^−7^	-	[135]

## Data Availability

Not Applicable.
